# Investigating the psammophilic karyorelictean ciliate families Kentrophoridae and Cryptopharyngidae (Protista, Ciliophora): molecular phylogeny, geographic distributions and a brief revision including descriptions of a new genus, a new species and a new combination

**DOI:** 10.1007/s42995-024-00266-6

**Published:** 2024-12-23

**Authors:** Mingzhen Ma, Danxu Tang, Wen Song, Lifang Li, Igor V. Dovgal, Khaled A. S. Al-Rasheid, Hunter N. Hines, Ying Yan

**Affiliations:** 1 Laboratory of Marine Protozoan Biodiversity & Evolution, Marine College, Shandong University, Weihai, 264209 China; 2 Laboratory of Protozoology, Institute of Evolution & Marine Biodiversity, and the Key Laboratory of Evolution & Marine Biodiversity (Ministry of Education), Ocean University of China, Qingdao, 266003 China; 3 A.O. Kovalevsky Institute of Biology of the Southern Seas, Sevastopol, 299011 Russia; 4 Zoology Department, College of Science, King Saud University, 11451 Riyadh, Saudi Arabia; 5 Harbor Branch Oceanographic Institute, Florida Atlantic University, Fort Pierce, FL 34946 USA

**Keywords:** *Cryptopharynx*, *Kentrophoros*, *Parakentrophoros*, Phylogeny, SSU rDNA

## Abstract

Psammophillic ciliates are an integral part of the foodweb despite being underrepresented in terms of molecular phylogeny and modern taxonomy. To investigate the karyorelictean group, sampling was conducted in interstitial marine habitats in China for ciliates living between the sand grains, resulting in an examination of the families Cryptopharyngidae Jankowski, 1980 and Kentrophoridae Jankowski, 1980. Three species, i.e., *Cryptopharynx setigerus* Kahl, 1928, *Kentrophoros fasciolatus* (Sauerbrey, 1928) Foissner, 1995 and *K. fistulosus* (Fauré-Fremiet, 1950) Foissner, 1995, are clearly recognized as being cosmopolitan, while other species await further recording. Phylogenetic analyses were carried out based on updated data. These revealed that the families Cryptopharyngidae and Kentrophoridae are closely related, and most genera studied are monophyletic, although *Cryptopharynx qingdaoensis* n. sp. is located within the Kentrophoridae branch. Brief revisions of two genera, namely *Cryptopharynx* Kahl, 1928 and *Kentrophoros* Sauerbrey, 1928, are provided including keys to the identification of nine species belonging to the former and 12 species belonging to the latter. One new genus, *Parakentrophoros* n. gen., and one new species, *Cryptopharynx qingdaoensis* n. sp., are described and a new combination, *Parakentrophoros canalis* (Wright, 1982) n. comb., is established. Finally, it appears that the subapical oral apparatus undergoes a gradual degeneration process from Cryptopharyngidae to Kentrophoridae.

## Introduction

The intertidal zone of coastal marine sandy shorelines contains some fascinating and unusual biotopes (Carey 1992; Chi et al. 2021; Hu et al. 2019; Liu et al. 2022; Song et al. 2009; Ye et al. 2022). Thriving between the sand grains in these niches are numerous ciliates, with many belonging to the class Karyorelictea Corliss, 1974. Mesopsammic karyorelicteans are characterized by their elongated cells, which can reach lengths of over 1 mm in some cases (Foissner 1995). If removed from the sand where they live, they can be easily observed with the naked eye. These cells have great importance as consumers in the interstitial food chain and for nutrient cycling of these sandy marine habitats (Fauré-Fremiet 1950; Finlay and Fenchel 1989; Kahl 1935; Ma et al. 2021a, b; Xu et al 2011a, b; Yan et al. 2013, 2015, 2016; Ye et al. 2023). Two predominant families from this habitat are Cryptopharyngidae Jankowski, 1980 (order Loxodida Jankowski, 1980) and Kentrophoridae Jankowski, 1980 (order Protostomatida Small & Lynn, 1985). Kentrophoridae Jankowski, 1980 is well-documented as possessing a “bacterial kitchen garden” living on its surface. These ectosymbionts serve a food source for their ciliate host (Edgcomb et al. 2011; Fenchel and Finlay 1989). The main feature of Cryptopharyngidae Jankowski, 1980 is its covering of distinct, ornamented scales embedded in a mucous layer on the left side. Most studies on these two families were carried out in the 2nd half of the twentieth century (Dragesco 1954a, b, 1965; Fauré-Fremiet 1950, 1951; Fenchel and Finlay 1989; Foissner 1995, 1996; Raikov 1962; Small and Lynn 1985; Wright 1982). In recent years, few taxonomic studies on these groups have been published (Bi et al. 2022; Seah et al. 2022; Xu et al. 2011a, 2017; Yan et al. 2017, 2019). Including new data from this study, there are currently ten nominal species of Cryptopharyngidae and 12 nominal species of Kentrophoridae. Among these the ciliature has been reported for only six cryptopharyngids and four kentrophorids. Therefore, these two families represent interesting groups for further research.

In the present study, one new genus and one new species are described, two rarely observed kentrophorid species are redescribed, and one new combination is established, based on a detailed modern morphological investigation and molecular phylogenetic analyses.

## Materials and methods

### Sample collection and morphological methods

Samples were collected from the intertidal zone of Qingdao, China, during ebb tides. A 20–30 cm diameter trough was excavated in the sand, and once the trough was filled with seawater, the top 5 cm of sand and seawater was collected. Ultimately, roughly 10 kg of combined seawater and sand was collected. In order to concentrate ciliates in the laboratory, a nylon gauze (pore size 80–90 μm) was used to filter the specimens. Initially, a 50 mm diameter and 100 mm long plastic tub was covered with nylon gauze at one end. About 30 mm of frozen fresh water and 30 mm of sand were positioned on the upper and lower layers, respectively, in the tube. The ciliates migrated downwards away from the advancing front of ice-melt. The filtered samples were collected into 60 mm diameter Petri dishes and then examined (Ma et al. 2022a, b; Uhlig 1964).

**Fig. 1:**
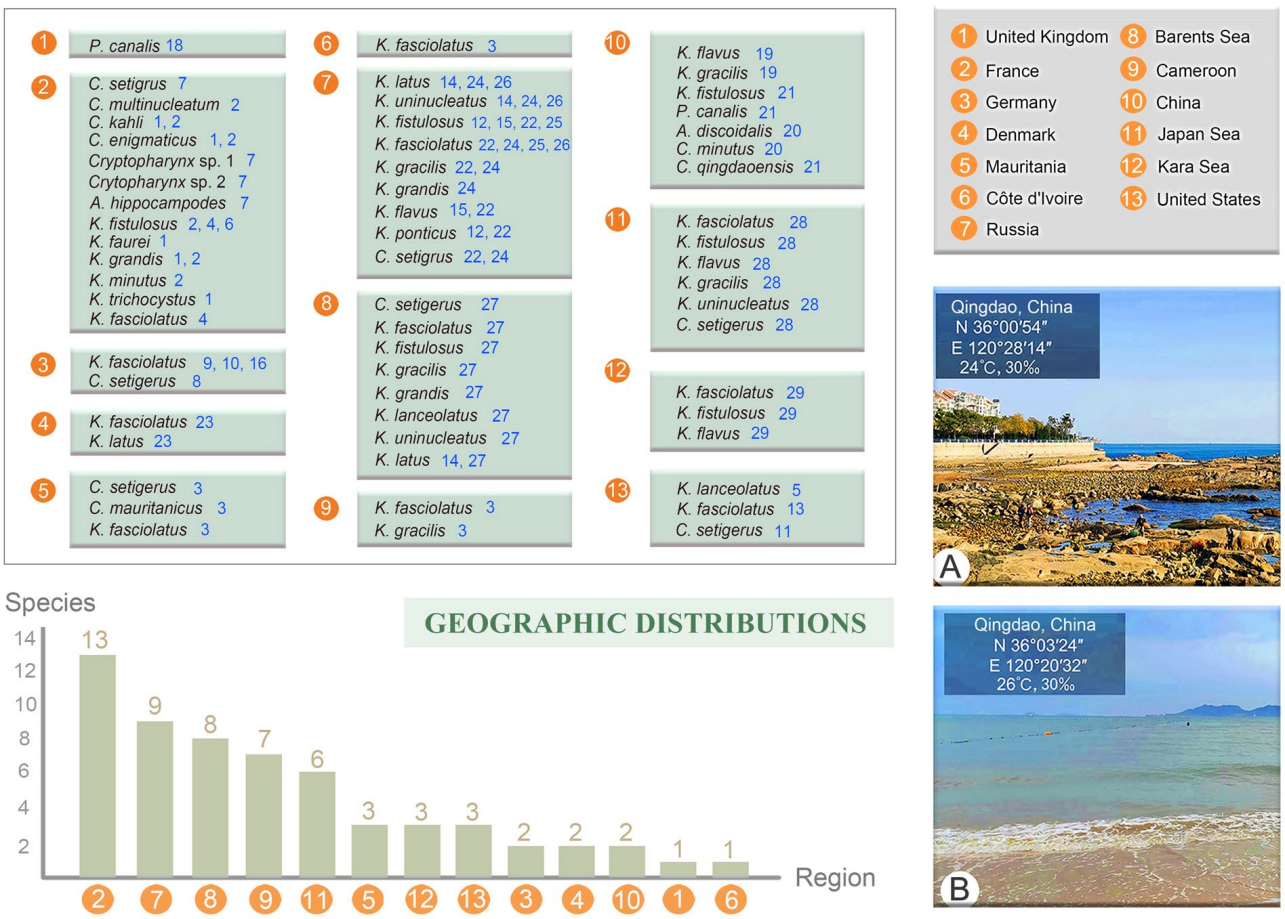
Global records of Cryptopharyngidae and Kentrophoridae species. Photographs show (**A**) the rocky intertidal zone and (**B**) of the sandy intertidal zone, sampled during this investigation. References are shown in blue numbers, and the details are as follows: 1. Dragesco (1954a, b), 2. Dragesco (1960), 3. Dragesco (1965), 4. Fauré-Fremiet (1950), 5. Fauré-Fremiet (1951), 6. Foissner (1995), 7. Foissner (1996), 8. Kahl (1928), 9. Kahl (1933), 10. Kahl (1935), 11. Kirby (1934), 12. Kovaleva (1966), 13. Noland (1937), 14. Raikov (1962), 15. Raikov and Kovaleva (1968), 16. Sauerbery (1928), 17. Small and Lynn (1985), 18. Wright (1982), 19. Xu et al. (2011a, b, c), 20. Xu et al. (2017), 21. present study, 22. Azovsky and Mazei (2003), 23. Fenchel and Finlay (1989), 24. Mazei and Burkovsky (2005), 25. Azovsky et al. (2013), 26. Li et al. (2023), 27. Azovsky and Mazei (2007), 28. Azovsky and Mazei (2013), 29. Azovsky and Mazei (2018), 30. Alekperov and Tahirova (2019)

*Cryptopharynx qingdaoensis* n. sp. was collected on June 24th, 2019, when the water temperature was 26 ºC, and the salinity 30‰. *Parakentrophoros canalis* (Wright, 1982) n. gen. n. comb. was collected on April 9th, 2013, when the water temperature was 20 ºC, and the salinity 30‰. *Kentrophoros fasciolatus* (Sauerbrey, 1928) Foissner, 1995 and *K. gracilis* (Raikov, 1963) Foissner, 1995 were collected on July 15th, 2019, when the water temperature was 24 ºC, and the salinity 30‰ (Fig. 1).

Cells were isolated with glass capillary pipettes. Live specimens were investigated using bright field and differential interference contrast microscopy at magnifications of 100 × to 1000 × using a Zeiss Axio Imager D2 microscope (Germany, Zeiss). Length measurements are taken when the ciliate was stationary and neither contracting nor elongating. The infraciliature was revealed with the protargol staining method according to Wilbert (1975). Measurements and counts were performed under 100–1000 × magnifications. Drawings of live specimens were based on living observations and photomicrographs. Drawings of stained specimens were made with a drawing device (Adobe Photoshop 2020). Terminology is according to Lynn (2008) and Xu et al. (2011a).

### DNA extraction, PCR amplification, and sequencing

For each species, a single cell was washed five times in sterilized sea water. Genomic DNA was extracted using the DNeasy Blood & Tissue Kit (Qiagen, Hilden, Germany) following the manufacturer’s instructions with the modification that 1/4 of the volume suggested for each reagent solution was used. The small subunit (SSU) rDNA sequence was amplified by PCR according to Xu et al. (2011b, c), using the primers: 18SF (5ʹ-AAC CTG GTT GAT CCT GCC AGT-3ʹ) (Medlin et al. 1988) and 5.8SR (5ʹ- TAC TGA TAT GCT TAA GTT CAG CGG-3ʹ) (Yi et al. 2009). Q5 Hot Start High-Fidelity DNA Polymerase (New England BioLabs, USA) was used for amplification. Direct sequencing of PCR products was performed by the Tsingke Biological Technology Company (Qingdao, China), using the original PCR primers and three additional primers (+ B: 5ʹ -GGTTAAAAAGCTCGTAGT-3ʹ; 900F: 5 ʹ-CGATCAGATACCGTCCTA GT -3ʹ; 900R: 5ʹ-ACTAGGACGGTATCTGATCG -3ʹ). The sequence fragments were assembled by Seqman v. 7. 1. 0.

### Phylogenetic analyses

**Fig. 2:**
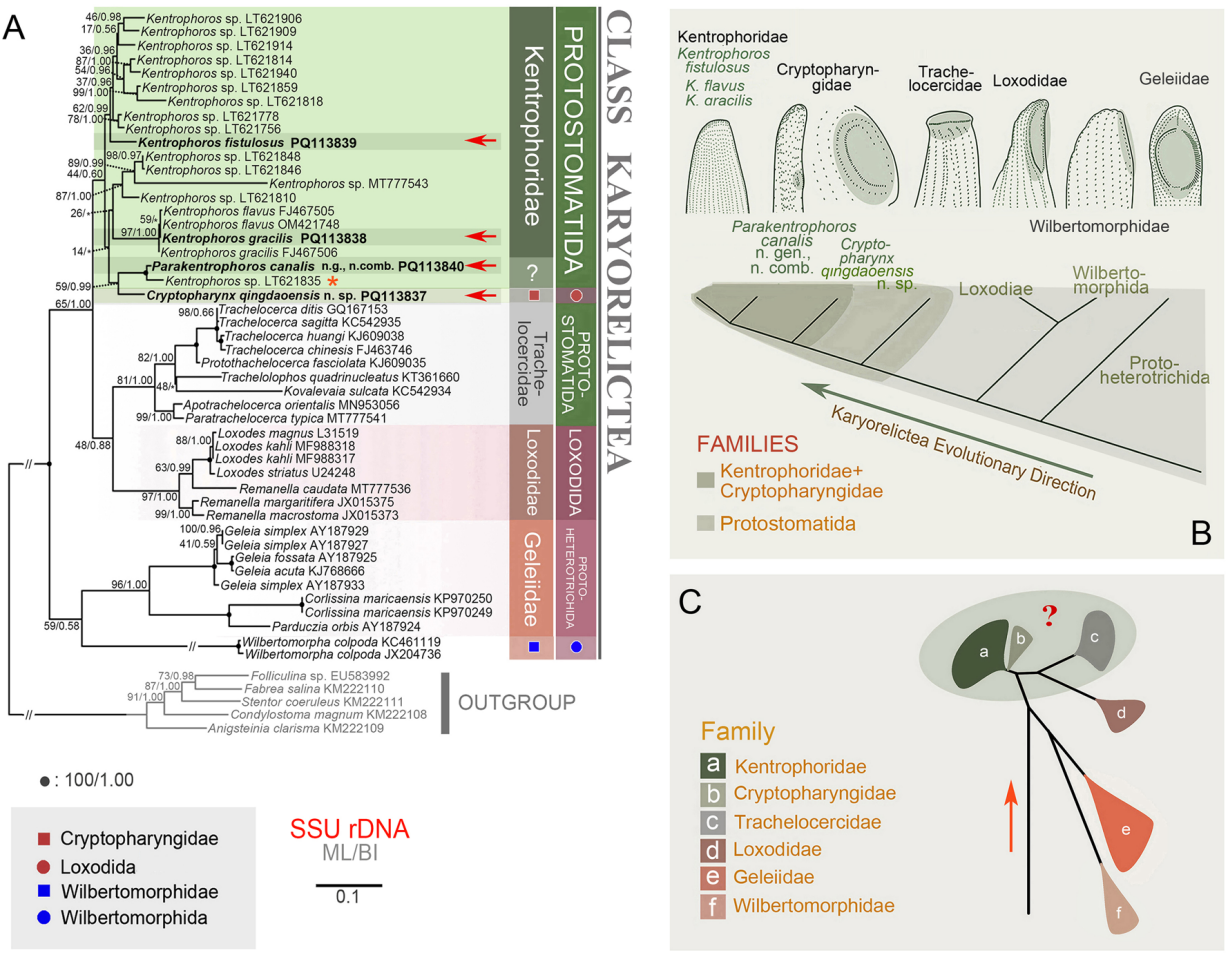
**A**. Maximum likelihood (ML) tree inferred from the SSU rDNA sequences showing the positions of the four newly sequenced Kentrophoridae and Cryptopharyngidae isolates (marked with red arrow heads). Nodal support for branches are marked in order of ML bootstrap and BI posterior probabilities. Black circles indicate full support in both analyses. Clades with a different topology between the two analyses are shown by an asterisk. Heterotrich species are the outgroup. The orange asterisk shows that this taxon is a misidentified, unknown species in the new genus *Parakentrophoros*. The scale bar corresponds to ten substitutions per 100 nucleotides positions. **B**. Oral ciliature of each genus in Karyorelictea using a phylogenetic framework. **C**. Showing a hypothetical evolutionary trajectory of families in the class Karyorelictea

Along with the four new sequences, another 43 sequences of karyorelicteans acquired from GenBank were used in the phylogenetic analyses. Five species of Heterotrichea (*Folliculina* sp. EU583992, *Fabrea salina* KM222110, *Stentor coeruleus* KM222111, *Condylostoma magnum* KM222108 and *Anigsteinia clarisma* KM222109) were selected as the outgroup taxa (Fig. 2). Sequences were aligned by the GUIDANCE2 algorithm online using the default parameters (Penn et al. 2010a, b). The resulting alignment was manually refined using BioEdit 7.0.5.2 (Hall 1999). The length of the final alignment for phylogenetic analyses was 1678 positions.

Maximum likelihood (ML) bootstrapping analysis was carried out with 1000 replicates, using RAxML-HPC2 v.8.2.10 on XSEDE (Stamatakis 2014) on CIPRES Science Gateway (http://www.phylo.org), with the GTRGAMMA model. Bayesian inference (BI) analysis was carried out using MrBayes v.3.2.6 on XSEDE (Ronquist et al. 2012) with the best fit model GTR + I + G selected by the Akaike Information Criterion (AIC) in MrModeltest 2.2 (Nylander 2004). The BI analysis was conducted with 1,000,000 generations and sampling every 100 generations. The first 25% of sampled trees were discarded as burn-in. Tree topologies were manually formatted with MEGA 7.0 (Kumar et al. 2016).

## Results

### Phylogenies inferred from SSU rDNA sequences (Fig. 2***)***

The topologies of the BI and ML trees were concordant therefore we present only the ML tree with node support from both algorithms. Four families, Trachelocercidae Kent, 1881, Loxodidae Bütschli, 1889, Geleiidae Kahl, 1933 and Wilbertomorphidae Xu et al., 2013, are monophyletic with moderate to high support (81% ML, 1.00 BI; 97% ML, 1.00 BI; 96% ML, 1.00 BI; 100% ML, 1.00 BI). However, Kentrophoridae is paraphyletic because Cryptopharyngidae grouped within it (Fig. 2A, C).

In GenBank, there are several *Kentrophoros* Sauerbrey, 1928 sequences that are not identified to species level*. Kentrophoros fistulosus* (Fauré-Fremiet, 1950) Foissner, 1995, with its tubular shaped body grouped with some *Kentrophoros* spp. with moderate support (62% ML, 1.00 BI). *Kentrophoros gracilis* (Raikov, 1963) Foissner, 1995 grouped with *K. flavus* Raikov & Kovaleva, 1968 with low support (59% ML) which together grouped with *K. gracilis* (FJ467506) with high support (97% ML, 1.00 BI). *Kentrophoros gracilis* (PQ113838) differs from *K. gracilis* FJ467506, *K. flavus* OM421748, and *K. flavus* FJ467505 by 2 bp, 3 bp and 4 bp, respectively. Although the sequences of *K. gracilis* (PQ113838) and *K. gracilis* FJ467506 differ the least, they did not cluster together. The sequence of *P. canalis* (Wright, 1982) n. gen. n. comb. (PQ113840) groups with *Kentrophoros* sp. LT621835 (100% ML, 1.00 BI), however there is a 28 bp difference between them. Cryptopharyngidae, represented by *Cryptopharynx qingdaoensis* n. sp. (PQ113837), is sequenced for the time. *Cryptopharynx qingdaoensis* clusters with *P. canalis* and *Kentrophoros* sp. LT621835 despite nucleotide differences of 80 bp and 76 bp respectively, within the Kentrophoridae assemblage, albeit with low support (44% ML, 0.60 BI) (Fig. 2A).

Class: Karyorelictea Corliss, 1974.

Order: Loxodida Jankowski, 1980.

Family: Cryptopharyngidae Jankowski, 1980.

Genus: *Cryptopharynx* Kahl, 1928

### ***Cryptopharynx qingdaoensis*** n. sp. (Figs. 3, 4; Table 1)

**Fig. 3:**
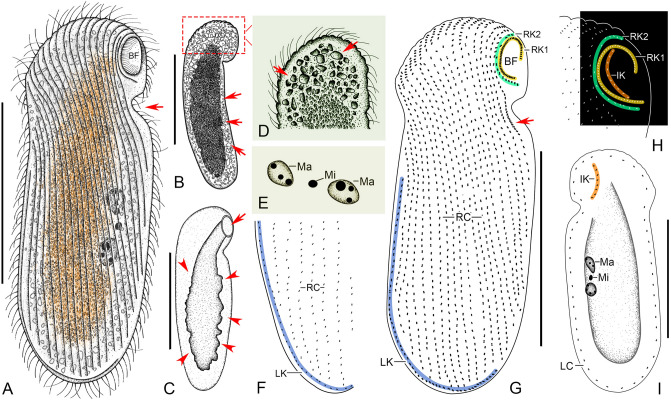
*Cryptopharynx qingdaoensis* n. sp. from life (**A**–**D**) and after protargol staining (**E**–**I**). **A** Right side of a representative specimen with arrow denoting the concavity under the buccal field. **B** Left side of a representative specimen. Arrows show irregular particles on the surface. **C** Outline of left side. Arrowheads show outline of thicker part on left side. Arrow shows buccal field. **D** Anterior of left side. Arrows show irregular particles on the surface. **E** Nuclear group. **F** Posterior of right infraciliature. **G** Infraciliature of right side of holotype specimen. Arrow marks the densely arranged kinetids below the buccal field. **H** Oral structure, showing first and second right buccal kineties, and intrabuccal kinety. **I** Infraciliature of left side of holotype specimen. *BF* buccal field, *IK* intrabuccal kinety, *LC* left ciliary row, *LK* dorsolateral kinety, *Ma* macronucleus, *Mi* micronucleus, *RC* right ciliary rows, *RK1* first right buccal kinety, *RK2* second right buccal kinety. Scale bars: 50 µm (**A**–**C**, **G**, **I**)

**Fig. 4:**
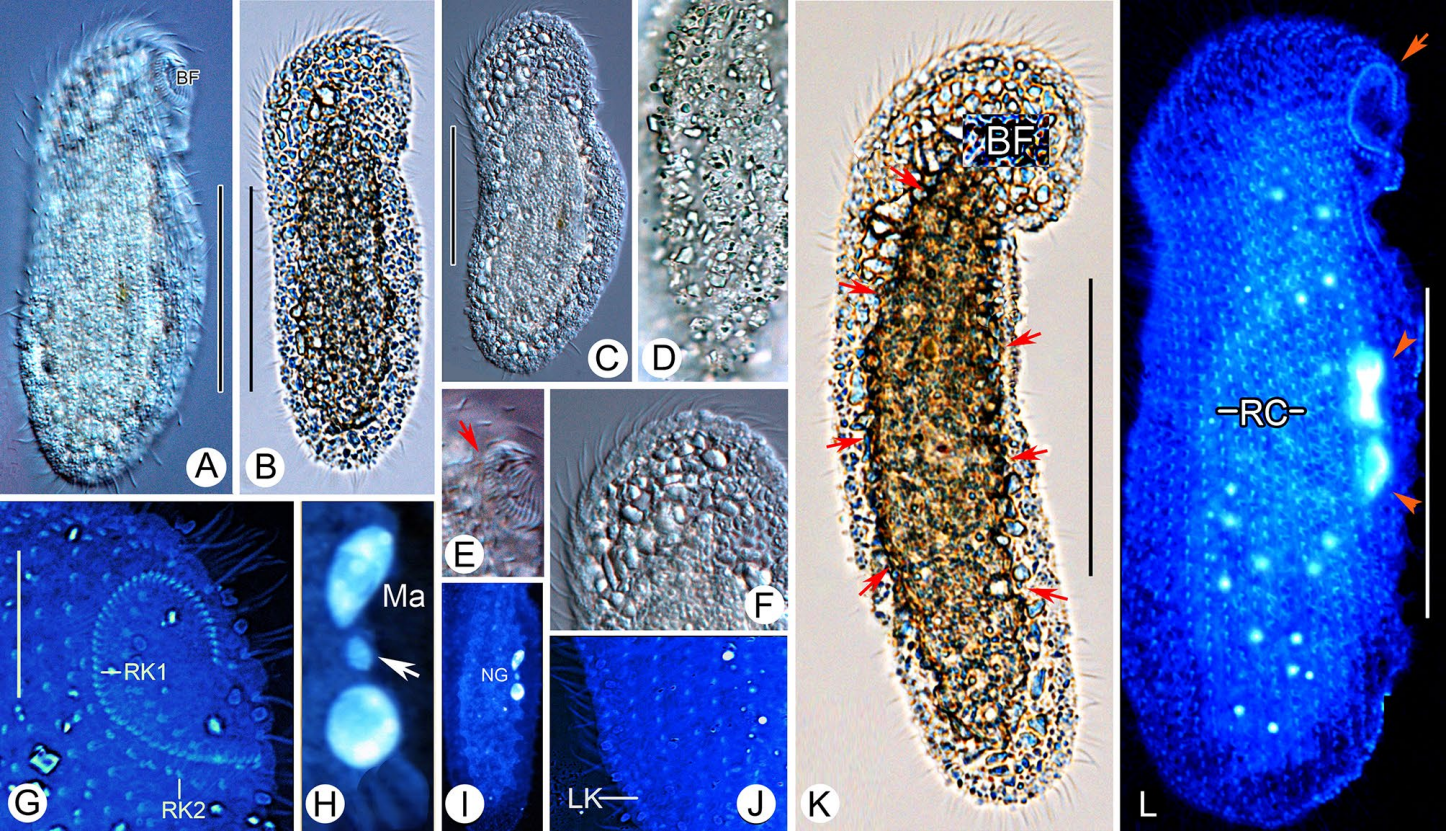
*Cryptopharynx qingdaoensis* n. sp. from life (**A**–**F**, **K**) and after protargol staining (**G**–**J**, **L**). **A** Right side of a representative specimen. **B**, **C**, **K** Left side of a representative specimen. Arrows show the edge of the raised center. **D** Left view, showing densely packed irregular particles on cell surface. **E** Buccal field. Arrows show buccal cilia. **F** Anterior of left side. **G** Oral structure, showing first and second right buccal kineties. **H** Nuclear group. Arrow shows micronucleus. **I** Outline of left side, thicker part. **J** Posterior of right infraciliature. **L** Infraciliature of right side of holotype specimen. *BF* buccal field, *LK* dorsolateral kinety, *Ma* macronuclei, *NG* nuclear group, *RC* right ciliary rows, *RK1* first right buccal kinety, *RK2* second right buccal kinety. Scale bars: 50 µm (**A**–**C**, **K**, **L**); 15 µm (**H**)

**Table 1:** Morphometric data for Qingdao populations of *Cryptopharynx qingdaoensis* n. sp. (*C. qin*), *Parakentrophoros canalis *(Wright, 1982) n. gen. n. comb. (*P. can*), *Kentrophoros fistulosus* (Fauré-Fremiet, 1950) Foissner, 1995 (*K. fis*), and *Kentrophoros gracilis* (Raikov, 1963) Foissner, 1995 (*K. gra*) from protargol-stained speciemens

Characters	species	Min	Max	Mean	SD	CV	n
Cell length (μm)	*C. qin*	75	110	89.8	11.9	13.2	13
	*P. can*	230	830	422.4	177.3	42.0	11
	*K. fis*	195	520	336.0	85.0	25.3	25
	*K. gra*	170	580	344.3	123.2	35.8	15
Cell width (μm)	*C. qin*	25	39	29.6	4.7	15.9	13
	*P. can*	23	60	37.5	11.2	29.8	11
	*K. fis*	40	100	73.0	12.7	17.4	25
	*K. gra*	15	25	21.0	3.9	18.4	15
Somatic kineties, number	*C. qin*	15	22	18.6	1.7	9.2	13
	*P. can*	9	12	10.1	1.0	9.8	8
	*K. fis*	23	40	29.6	5.0	16.7	23
	*K. gra*	8	11	9.6	0.7	7.3	25
Macronuclei, number	*C. qin*	1	2	1.9	0.3	14.4	13
	*P. can*	2	6	4.7	1.2	25.2	11
	*K. fis*	1	4	3.2	1.1	35.1	25
	*K. gra*	6	19	11.9	3.5	29.4	25
Micronuclei, number	*C. qin*	1	1	1.0	0	0	13
	*P. can*	2	2	2.0	0	0	11
	*K. fis*	1	2	1.7	0.5	28.3	25
	*K. gra*	4	13	8.4	2.9	34.2	25
Nuclear groups, number	*K. fis*	2	20	7.7	4.3	56.2	26
Dikinetids in first right buccal kinety, number	*C. qin*	30	54	44.3	8.2	18.6	12
Dikinetids in second right buccal kinety, number	*C. qin*	9	13	11.9	1.4	11.6	12
Dikinetids in intrabuccal kinety, number	*C. qin*	7	12	9.5	1.5	15.3	13

*Min* minimum, *Max* maximum, *Mean* arithmetic mean, *SD* standard deviation of the mean, *CV* coefficient of variation in %, *n* number of specimens investigated

**Diagnosis.** Cell in vivo about 80–120 × 25–40 µm, oval discoid, oral area protruding. Cytoplasm colorless or brownish; left lateral surface covered with irregular particles. Protrusion in mid-left region, cell margins concaved. 15–22 right ciliary rows. First and second right buccal kinety and intrabuccal kinety composed of 30–54, 9–13 and 7–12 dikinetids, respectively. Nuclear group near center of cell consisting of 1–2 macronuclei and one micronucleus.

**Type locality.** The intertidal zone of No. 1 Bathing Beach located at Qingdao (36°03′24″N, 120°20′32″E), China (Fig. 1B).

**Deposition of slides.** A protargol slide containing the holotype specimen marked with an inked circle has been deposited at the Laboratory of Protozoology, OUC, China (No. MMZ2019062403).

**Etymology.** The species group name *qingdaoensis* means the species was originally isolated from Qingdao.

**Description.** Cell in vivo about 80–120 × 25–40 µm, usually 90 × 30 µm, oval discoid, laterally flattened, mid-left region thicker than cell margins, right side flat (Figs. 3A–C, 4A–C, K). Oral area protruding forwards, located near anterior end of cell (Figs. 3C, 4E). Left surface covered with numerous irregular particles (Figs. 3D, 4D). Somatic cilia about 10 µm long. Colorless oval particles 1 × 2 µm, probably cortical granules, densely scattered in mid-left mid region and between right ciliary rows (Figs. 3B, D, 4C, F). Locomotion by crawling over substrate at moderate speed, sometimes sticking tightly to surface.

Fifteen to 22 right ciliary rows, composed of dikinetids and aligned approximately in parallel (Figs. 3F, G, 4L). Buccal overture elliptical. Oral ciliature composed of first and second right buccal kineties and intrabuccal kinety (Figs. 3G, H, 4G, L). First right buccal kinety composed of 30–54 densely packed dikinetids, extending along right and anterior margin of buccal overture, curving backward along left overture margin, terminating at middle of buccal area. Second right buccal kinety composed of 9–13 dikinetids, terminating in apical region of cell. Intrabuccal kinety composed of 7–12 dikinetids, extending into buccal cavity. Dorsolateral kinety extending along posterior cell margin to posterior end cell (Figs. 3F, G, 4J). Left ciliary row extending around cell (Fig. 3I). Nuclear group located in mid-region of cell, composed of 1–2 macronuclei and one micronucleus (Figs. 3E, 4H, I).

Order: Protostomatida Small & Lynn, 1985.

Family: Cryptopharyngidae Jankowski, 1980.

Genus: *Parakentrophoros* n. gen.

**Diagnosis.** Kentrophoridae with densely packed epibiontic bacteria that cover entire cell surface apart from central stripe on right side. Several dikinetids grouped together and located subapically; somatic kineties on right side and densely arranged in a band-like region down body length.

**Type species.** *Parakentrophoros canalis* (Wright, 1982) n. gen. n. comb.

### ***Parakentrophoros canalis*** (Wright, 1982) n. gen. n. comb. (Figs. 5, 6***; ***Table 1***)***

**Fig. 5:**
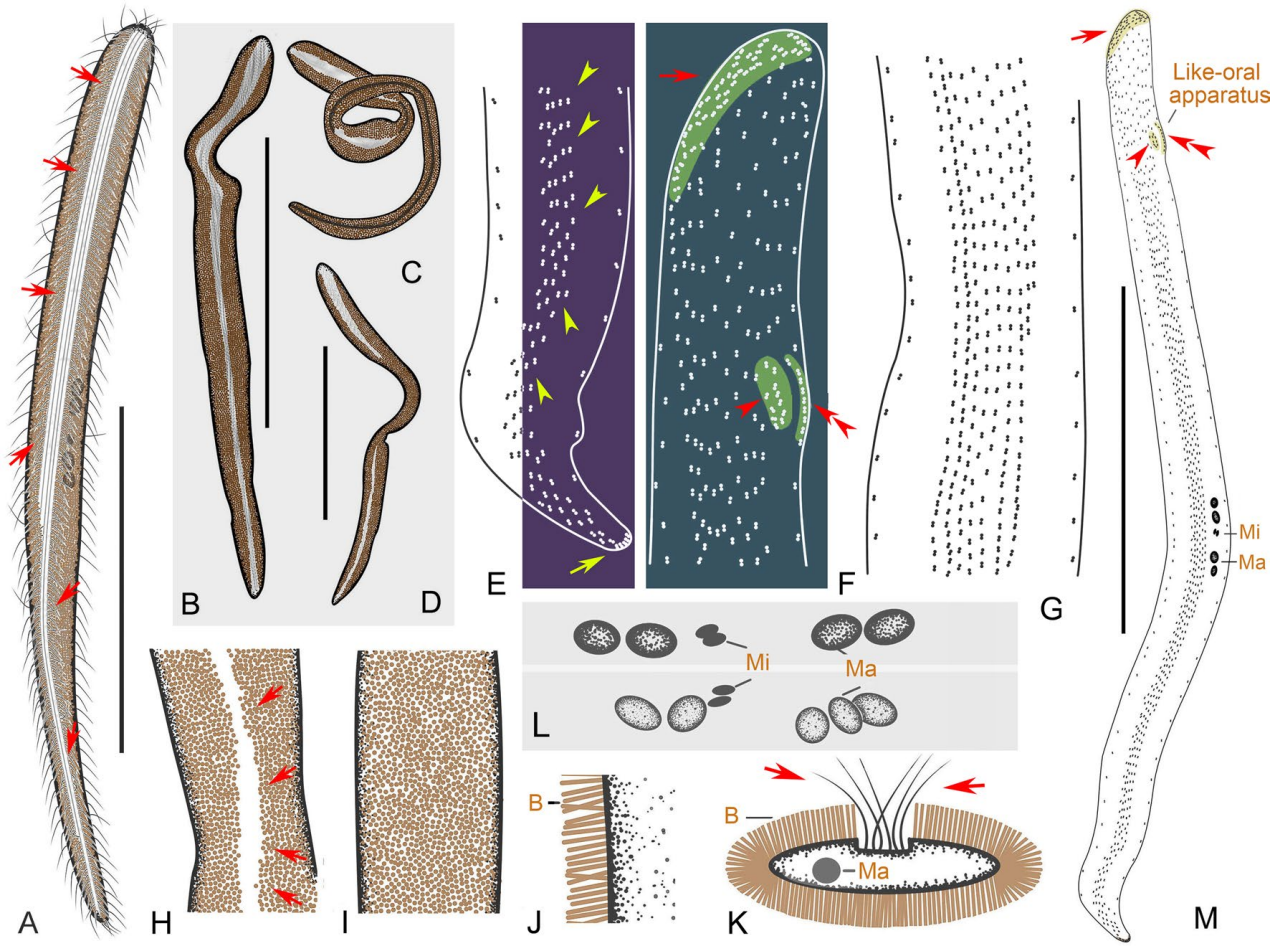
*Parakentrophoros canalis* from life (**A**–**D**, **H**–**K**) and after protargol staining (**E**–**G**, **L**, **M**). **A** Right view of typical individual. Arrows show right cilia. **B**–**D** Contorted individuals showing cell flexibility. **E** Posterior cell end in right lateral view to show right ciliary rows. Arrow marks the condensed dikinetids. Arrowheads indicate right ciliary rows. **F** Anterior end in right lateral view to show right ciliary rows. Arrow shows the obliquely oriented anteriormost dikinetids. Arrowhead shows irregularly arranged dikinetids. Double arrowheads show like-right buccal kinety. **G** Right view of infraciliature in mid-cell region. **H** Bacteria on right side. Arrows show bacteria. **I** Bacteria on left side. **J** The epibiontic bacteria attached to the cell surface. **K** Transverse section. Arrows show somatic cilia. **L** Nuclear groups. **M** Right view showing the infraciliature and nuclear group. Arrow shows the obliquely oriented anteriormost dikinetids. Arrowhead shows irregularly arranged dikinetids. Double arrowheads show like right buccal kinety. *B* bacteria, *Ma* macronuclei, *Mi* micronuclei. Scale bar: 200 µm (**A**–**D**); 300 µm (**M**)

**Fig. 6:**
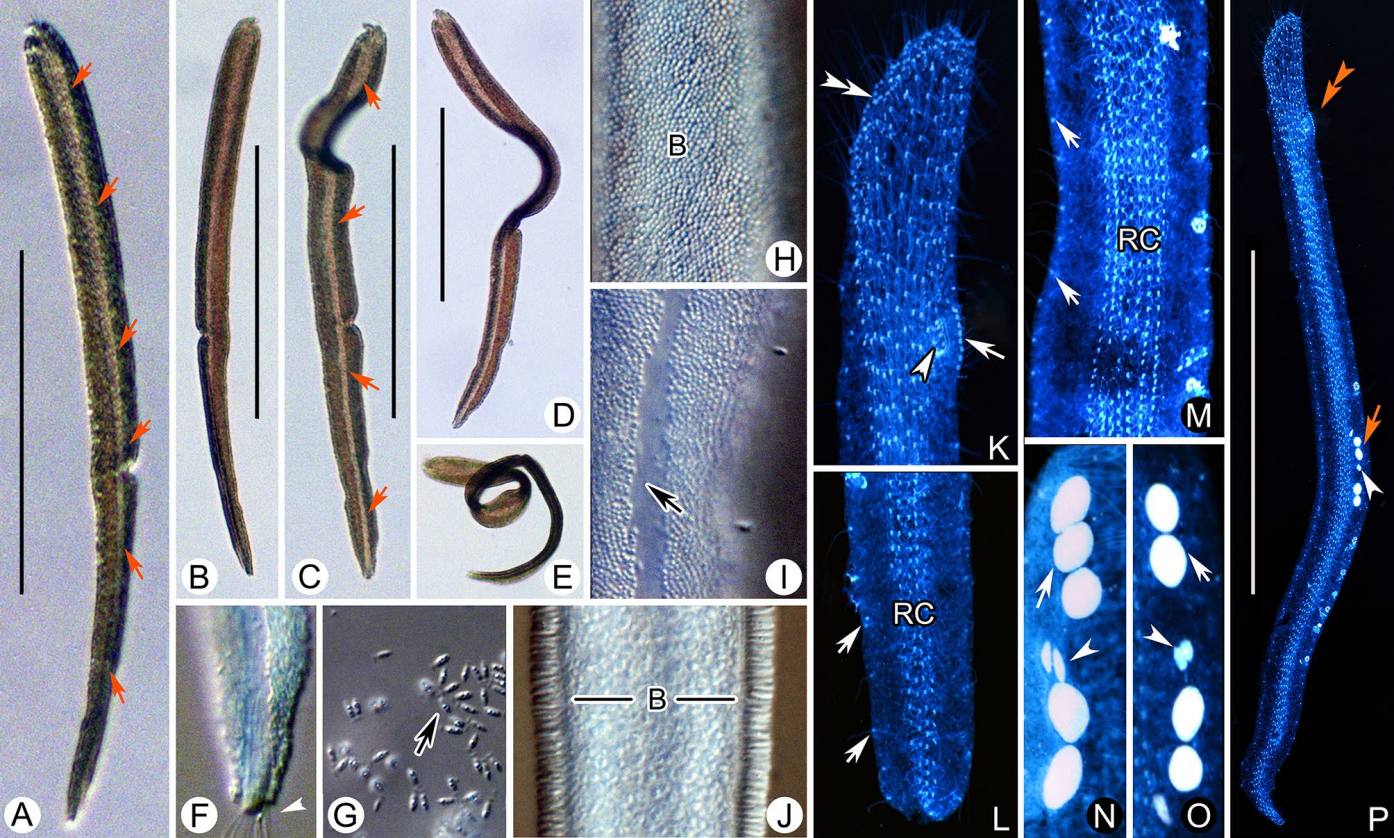
*Parakentrophoros canalis* from life (**A**–**J**) and after protargol staining (**K**–**P**). **A** Right view of a typical individual. Arrows show right ciliary rows. **B**–**E** Contorted individuals showing cell flexibility. Arrows show right ciliary rows. **F** Posterior region of right side. Arrow shows posterior end. **G** The epibiontic bacteria (arrow). **H** Bacteria on left side. **I** Bacteria on right side. Arrow shows middle unciliated part. **J** Marginal mid-cell region of right side. **K** Anterior end in right lateral view to show right ciliary rows. Arrow shows like-right buccal kinety. Double arrowheads show the obliquely oriented anteriormost dikinetids. Arrowhead shows irregularly arranged dikinetids. **L** Right lateral view of posterior end. Arrows show left ciliary row. **M** Right view of infraciliature in mid-region of cell. Arrows show left ciliary row. **N**, **O**. Nuclear groups. Arrows show macronucleus. Arrowheads show micronucleus. **P** Right view showing the infraciliature and nuclear group. Arrow shows macronucleus. Arrowhead shows micronucleus. *B* bacteria, *RC* right ciliary rows. Scale bar: 200 µm (**A**–**D**); 300 µm (**P**)

Basonym: *Kentrophoros canalis* Wright, 1982.

The original description of *Parakentrophoros canalis* (Wright, 1982) n. gen. n. comb. was based solely on observations of specimens in vivo (Wright 1982). Later, a population from the Caspian Sea was reported by Alekperov and Tahirova (2019) but without further details of its morphology. Hence, the infraciliature of this species has never been reported and its circumscription remains unclear. Here, based on the Qingdao population, a redescription and improved diagnosis are supplied.

**Improved diagnosis.** Cell in vivo about 300–850 × 25–30 µm; highly flattened about 600 × 25 μm in vivo. 2–6 macronuclei (usually 5) and 2 micronuclei arranged in an irregular row. About 9–12 somatic kineties on anterior right side, 3–4 somatic kineties on posterior right side. Densely packed epibiontic bacteria covering entire cell surface apart from a central stripe on right side.

**Description of Chinese population.** Cell size in vivo usually about 600 × 25 μm, moderately contractile and flexible (Figs. 5A–D, 6A–E). The anterior end blunter than the posterior. Cell length to width ratio about 30:1 when fully extended, about 10:1 when contracted (Figs. 5A–D, 6A–E). In incident light, cell is opaque due to the dense lawn of epibiontic bacteria (each cell about 3 × 0.8 μm) covering the entire cell surface apart from a central stripe on right side that runs entire length of cell which is bacteria-free and is lighter in appearance (Figs. 5A–D, H–K, 6A–J). Somatic cilia about 7 μm long in vivo. Neither food vacuoles nor contractile vacuoles observed. Locomotion by gliding on bottom of Petri dish, usually attaching to substrate when disturbed.

Nine to 12 longitudinal somatic kineties on anterior right side and 3–4 somatic kineties on posterior right side, composed of dikinetids throughout (Figs. 5E–G, M, 6K–M, P). Dikinetids more closely spaced in anterior region. Marginal dikinetids at posterior end of cell densely arranged. On the anterior right margin, Like-oral apparatus consisting of 8–11 closely spaced dikinetids (Figs. 5F, 6K). Several irregularly arranged dikinetids near the like-right buccal kinety, but it was not possible to determine whether these are barren or ciliated (Figs. 5F, 6K). On left side, one circle kinety surrounding cell margin. Nuclear apparatus forming a longitudinally oriented strand along cell margin, composed of 2–6 macronuclei, each about 4 μm in diameter, and two micronuclei; nuclei mostly globular or ellipsoidal (Figs. 5L, 6N, O).

### ***Kentrophoros fistulosus ***(Fauré-Fremiet, 1950) Foissner, 1995 (Figs. 7, 8; Table 1)

**Fig. 7:**
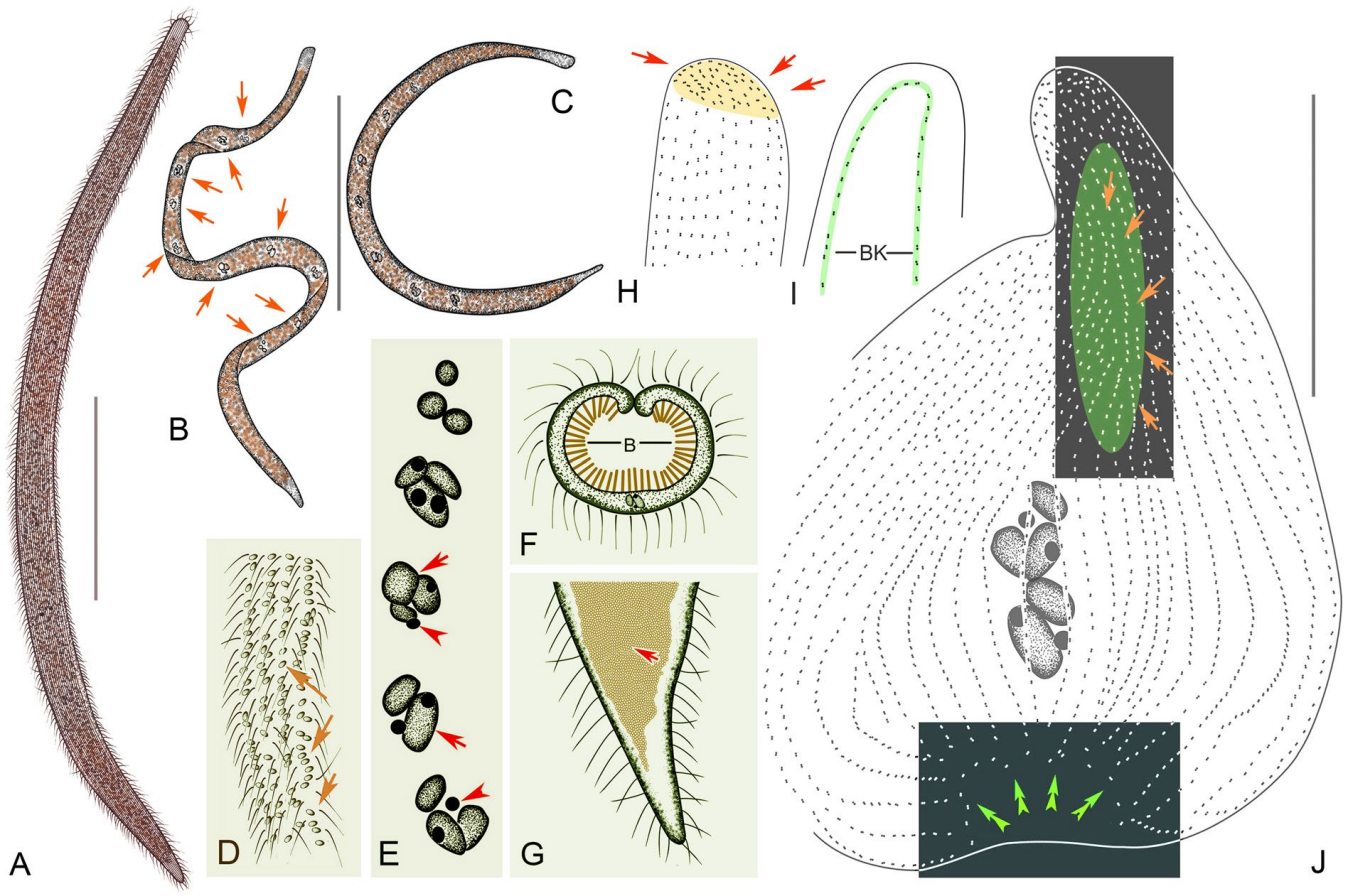
*Kentrophoros fistulosus* from life (**A**–**D**, **F**, **G**) and after protargol staining (**E**, **H**–**J**). **A** View of typical individual. **B**, **C** Contorted individuals. **D** Surface view showing cortical granules (arrows). **E** Nuclear groups. Arrows show macronuclei. Arrowheads show micronuclei. Right and left lateral view of anterior cell region. Arrows show the obliquely oriented anteriormost dikinetids. **F** Transverse section. **G** Posterior region of left side. Arrow shows bacteria. **H**, **I** Right and left lateral view of anterior cell region. Arrows show the obliquely oriented anteriormost dikinetids. **J** Right view showing the infraciliature and nuclear group. Arrows show left ciliary row. Double arrowheads show the left lateral ciliary row. Green part shows chaotic right ciliary rows. *B* bacteria; *LC* left lateral ciliary row. Scale bars: 300 µm (**A**–**C**); 150 µm (**J**)

**Fig. 8:**
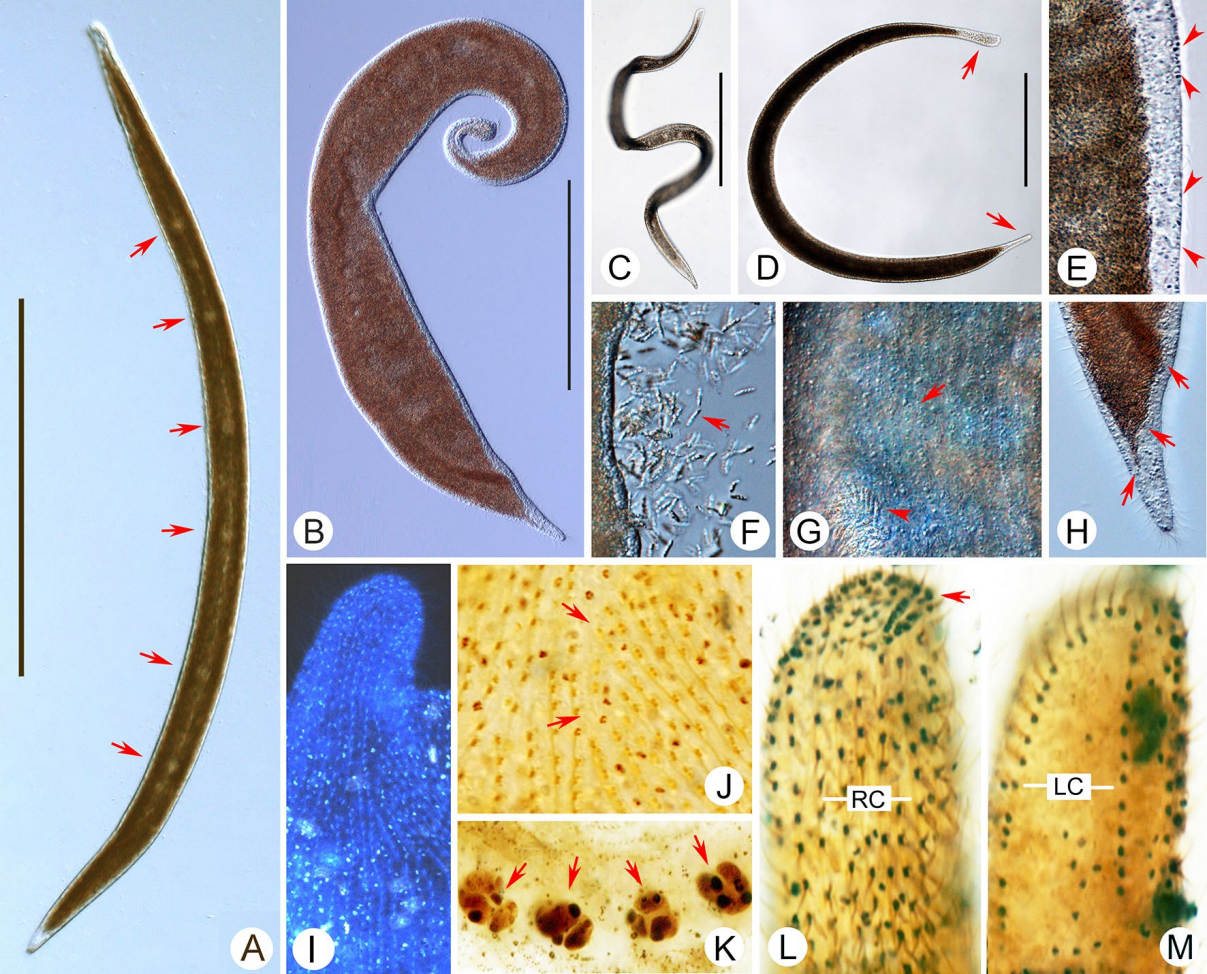
*Kentrophoros fistulosus* from life (**A**–**H**) and after protargol staining (**I**–**M**). **A** View of typical individual. Arrows show nuclear apparatus. **B**–**D**. Contorted individuals. Arrows show transparent anterior and posterior. **E** Left lateral view of margin of mid-region of cell. Arrowheads show cortical granules. **F** The epibiontic bacteria (arrow). **G** Right lateral view of middle region. Arrow shows cortical granules. Arrowhead shows epibiontic bacteria. **H** Left lateral view of posterior region. Arrows show epibiontic bacteria. **I** Right lateral view of anterior cell region showing chaotic right cilia rows. **J** Right lateral view of showing chaotic right cilia rows (arrows). **K** Nuclear groups (arrows). **L**, **M** Right and left lateral view of anterior cell region. Arrow shows the obliquely oriented anteriormost dikinetids. *RC* right lateral ciliary row; *LC* left lateral ciliary row. Scale bar: 500 µm (**A**); 300 µm (**B**, **C**); 200 µm (**D**)

*Kentrophoros fistulosus* was originally described by Fauré-Fremiet (1950) under the name *Centrophorella fistulosa*. Dragesco (1960) reported *Centrophorella fistulosa* along with a drawing. Foissner suggested that *Centrophorella* is a junior synonym of *Kentrophoros* Sauerbrey, 1928. Considering the genus and species gender, Foissner (1995) established a new combination for this species, named *Kentrophoros fistulosus* (Foissner 1995), and provided details of its morphometric data and ultrastructure. An improved diagnosis based on the present and previous populations is provided here. Furthermore, the first sequence for this species was obtained during this study.

**Improved diagnosis.** Cell about 400–2800 × 20–50 μm in vivo. 2–34 nuclear groups arranged along the midline of the trunk, each composed of 2–9 macronuclei and 1–4 micronuclei. About 9–12 somatic kineties on anterior right side, and 23–43 somatic kineties on remainder of right side. Black cell portion tube-shaped with covering of densely packed epibiontic bacteria on left side which forms inner surface of tube, ciliated right surface forming outer surface of tube. Cortical granules globular and colorless, about 1 μm in diameter.

**Description of Chinese population.** Size in vivo about 400–1500 × 30–50 μm; very slender and filiform, central region involuted forming a tube; cell black except for anterior part and narrowed posterior region (Figs. 7A–C, 8A–D). Cell length to width ratio about 30–70: 1 when fully extended. Black cell portion tubular with layer of epibiontic bacteria (about 1 × 5 μm) on inside surface of tubular region (left side), and ciliated on outside surface of tubular region (right side) (Figs. 7H, I, 8E, F, H). Somatic cilia about 10 μm long in vivo. Cytoplasm colorless, mostly transparent. Neither food vacuoles nor contractile vacuoles observed. Cortical granules globular and colorless, about 1 μm in diameter (Figs. 7F, 8G). Locomotion by gliding on bottom of Petri dish, usually attaching to substrate when disturbed.

Twenty-three to 40 longitudinal somatic kineties on right side composed of dikinetids throughout (Figs. 7J, 8I, J). Dikinetids more closely spaced at anterior end (Figs. 7D, 8L). On left side, one circle kinety surrounds cell margin (Figs. 7E, 8M). Two to 20 nuclear groups forming a longitudinally oriented strand along cell meridian, mostly composed of 2–4 macronuclei, each about 4 μm in diameter, and 1–2 micronuclei (Figs. 7G, 8K). Nuclei mostly globular or ellipsoidal.

### ***Kentrophoros gracilis ***(Raikov, 1963) Foissner, 1995 (Figs. 9, 10; Table 1)

**Fig. 9:**
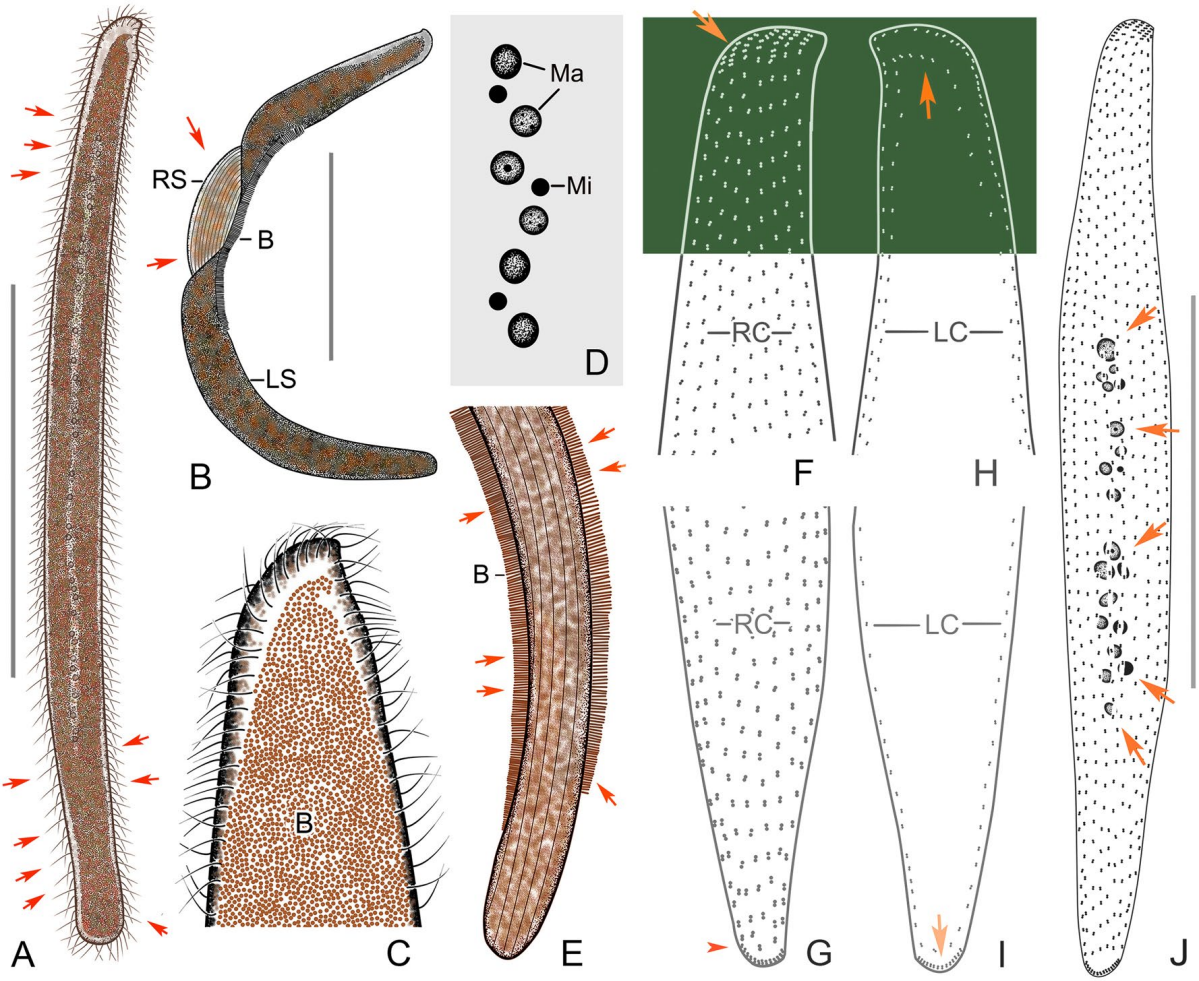
*Kentrophoros flavus* from life (**A**–**C**, **E**) and after protargol staining (**D**, **F**–**J**). **A** Left view of typical individual. Arrows show left cilia. **B** A contorted individual. Arrows show the right side. **C** Anterior end of left side. **D** Nuclear group. **E** Posterior end of right side. Arrows show the epibiontic bacteria. **F**–**I**. Anterior and posterior ends of cell in right and left lateral view to show right and left ciliary rows. Arrow in F marks the obliquely oriented anteriormost dikinetids. Arrowhead in G shows distinctly condensed dikinetids in posterior region. Arrow in H indicates left ciliary row. Arrow in I shows distinctly condensed dikinetids in posterior region. J. Right view showing the infraciliature and nuclear groups (arrows). *B* bacteria; *LC*, left ciliary row; *LS* left side; *Ma* macronuclei; *Mi* micronuclei; *RC* right ciliary rows; *RS* right side. Scale bar: 200 µm (**A**, **B**, **J**)

**Fig. 10:**
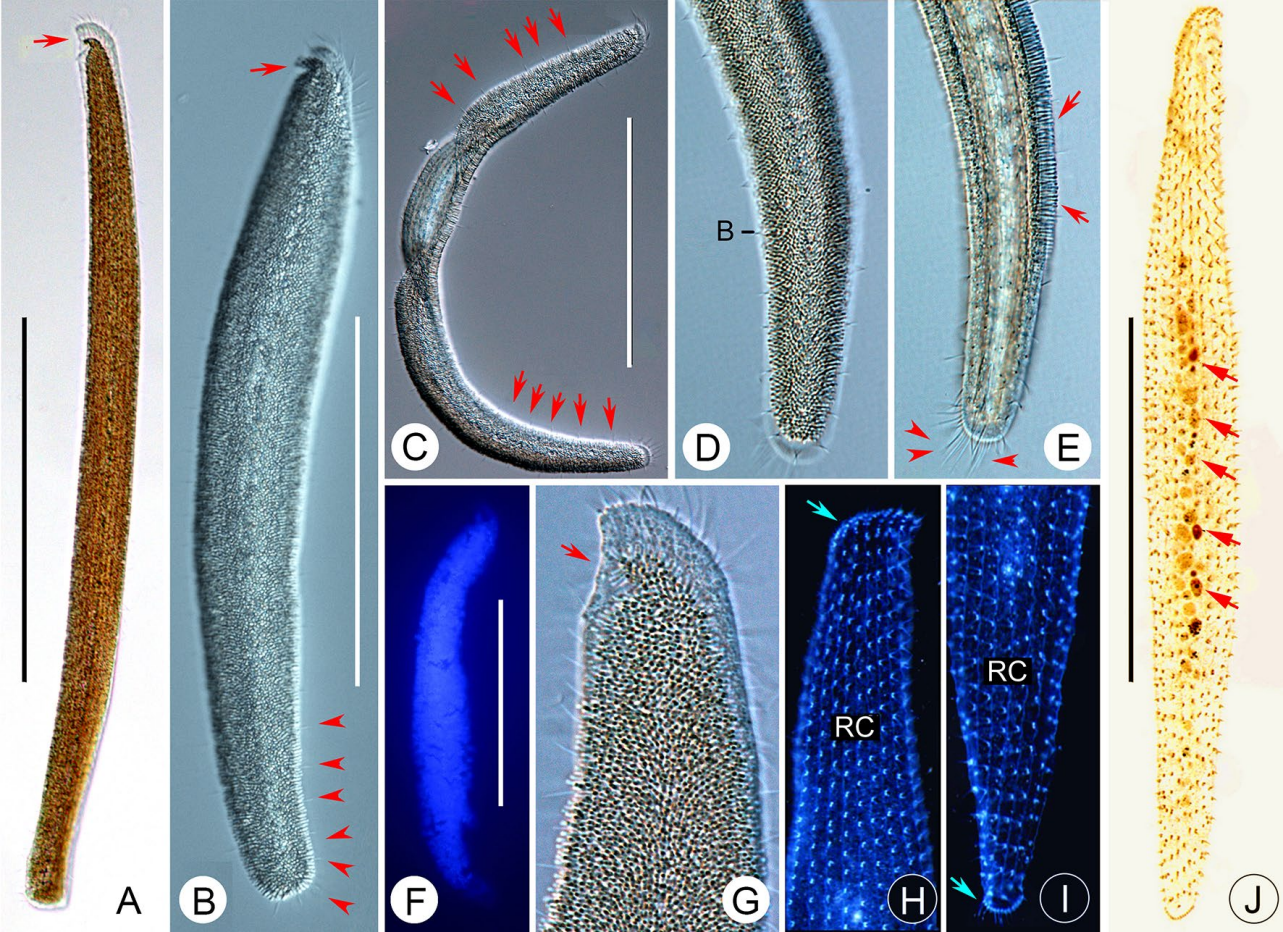
*Kentrophoros flavus* from life (**A**–**E**, **G**), after DAPI staining (**F**), and after protargol staining (**H**–**J**). **A** Left view of typical individual. Arrow shows anterior concavity. **B**, **C**. Contorted individuals. Arrow in B shows anterior concavity. Arrowheads in B show left cilia. Arrows in C show left cilia. **D** Posterior portion of left side. **E** Posterior portion of right side. Arrows show the epibiontic bacteria. Arrowheads show left cilia. **F** DAPI-stained cell showing epibiontic bacteria. **G** Anterior portion of left side. Arrow shows anterior concavity. **H** Ciliary rows on anterior portion of right side. Arrow indicates the obliquely oriented anteriormost dikinetids of right side. **I** Ciliary rows on posterior portion of right side. Arrow indicates distinctly condensed dikinetids in posterior. **J** Right view showing the infraciliature and nuclear groups (arrows). *B* bacteria; *RC* right ciliary rows. Scale bar: 200 µm (**A**–**C**, **J**)

This species was first recorded by Raikov (1963) as *Kentrophoros graciles*. It was later reported by Dragesco (1965) under the name *Kentrophoros gracilis*. Foissner (1995) determined the species name to be *Kentrophoros gracilis*. Later it was redescribed by Xu et al. (2011a) with details of its infraciliature and morphometric data. Therefore, a simple improved diagnosis based on the present and previous populations is supplied here.

**Improved diagnosis.** Cell about 150–600 × 20–70 µm in vivo. flattened and ribbon-like. 7–25 macronuclei and 4–21 micronuclei arranged along the cell meridian. 8–13 ciliary rows on right side of cell. Densely arranged epibiontic bacteria covering left side of cell except for anterior end.

**Redescription.** Cell mostly about 200–400 × 20–40 µm in vivo, flexible and slightly contractile, flattened and ribbon-like with both ends broadly rounded (Figs. 9A, B, 10A–C). Anterior part slightly curved and forming a short rostrum. Cell often slightly brownish at low magnifications, colorless or grayish at high magnifications. Left side of cell except anterior end covered by rod-like epibiontic bacteria, 6 × 1 µm in size, that are arranged orthogonal to cell surface (Figs. 9C, E, 10D–G). Bright longitudinal strand along cell meridian caused by nuclear apparatus which comprises 6–19 macronuclei and 4–13 micronuclei arranged in a longitudinal row; macronuclei globular, about 5 µm across, micronuclei about 1–2 µm in diameter (Figs. 9A, D, J, 10A, B, J). No food vacuoles or contractile vacuoles observed. Locomotion by sluggish gliding on bottom of Petri dish.

**Infraciliature dikinetid.** About 8–11 ciliary rows on right side (Figs. 9F, G, J, 10H–J). Most somatic kineties loosely arranged with their axes parallel to main cell axis, although those at the anterior end are more closely spaced with axes oriented obliquely to the main cell axis (Figs. 9F, J, 10H, J). On left side, one circle kinety surrounds cell margin (Fig. 9H, I).

## Discussion

### Biogeographic distribution

The spatial distribution patterns as described in the literature for species in the families Cryptopharyngidae and Kentrophoridae are shown in Fig. 1. This summary is based on morphometry, which makes more precise identifications possible. Due to the previous and increasing amount of literature providing detailed morphometrics, numerous investigations from habitats world-wide provide reliable material upon which to map species distributions (Fig. 1). Because records of Cryptopharyngidae species are rare, it is less commonly reported globally, with most records coming from France, Russia, China, Mauritania, Germany, Capsian Sea, Barents Sea, Japan Sea and the USA (Alekperov and Tahirova 2019; Azovsky and Mazei 2003a, 2007; Dragesco 1954a, b, 1960, 1965; Foissner 1996; Kahl 1928; Kirby 1934; Xu et al. 2017). Reports of Kentrophoridae are more common than Cryptopharyngidae, so kentrophorids are more widely reported in the literature, and therefore appear more widely distributed. Kentrophoridae have been reported from diverse marine habitats and, in addition to those localities listed for Cryptopharyngidae, have also been recorded from Côte d'Ivoire, Denmark, Cameroon, Kara Sea and the United Kingdom (Azovsky and Mazei 2003a; Fauré-Fremiet 1950, 1951; Foissner 1995; Kahl 1935; Kovaleva 1966; Raikov 1962; Raikov and Kovaleva 1968; Sauerbery 1928; Small and Lynn 1985; Wright 1982; Xu et al. 2011a). The records of these two families are mainly concentrated in France, Russia, Barents Sea and Capsian Sea where 13, 9, 8 and 8 species have been reported, respectively (Fig. 1). This pattern is almost certainly due to the fact that historically there has been more researchers in Russia and Europe investigating these organisms resulting in more comprehensive coverage of these two families in these regions. In the future, more extensive surveys of these and other interesting ciliate groups are needed to obtain a broader understanding of the patterns of ciliate biogeographic distribution. It is widely accepted that increased sampling and research often reveals expansions of biogeographic ranges to any areas where the preferred ecological niche of a given ciliate species exists and is subsequently sampled (Madrid et al. 2023).

### Genus ***Cryptopharynx ***Kahl, 1928

The genus *Cryptopharynx* was first reported by Kahl (1928). Subsequently, Kirby (1934) re-described the type species *C. setigerus* Kahl, 1928. Dragesco (1960, 1965) reported several new species based on live observations. Small and Lynn (1985) provided details of the ciliature of *C. wardi*, which was later transferred to the genus *Apocryptopharynx* Foissner, 1996 based on the presence of a long, clip-shaped intrabuccal kinety (Foissner 1996). This species has yet to be sequenced. Carey (1992) produced a review of marine interstitial ciliates. However, Foissner (1995) was the first to show photomicrographs and diagrams of cryptopharyngids that include details of the infraciliature and other taxonomically informative morphological features, and also established the genus *Apocryptopharynx*. Currently, there are nine nominal species assigned to this genus including two unnamed species. Among these *C. setigerus*, *Cryptopharynx* sp. 1, *Cryptopharynx* sp. 2, *C. qingdaoensis* n. sp. and *C. minutus* Xu et al., 2011 have been described using protargol staining to reveal the ciliary pattern, with the rest described solely from observations of specimens in vivo. Here we present a description of a new species of *Cryptopharynx*, namely *C. qingdaoensis* n. sp., the first deposited molecular sequence of *Cryptopharynx* (belonging to *C. qingdaoensis* n. sp., Fig. 2), a revision of the genus *Cryptopharynx,* and a key to the identification of its species.

### Key to species of ***Cryptopharynx ***(Fig. 11)

**Fig. 11:**
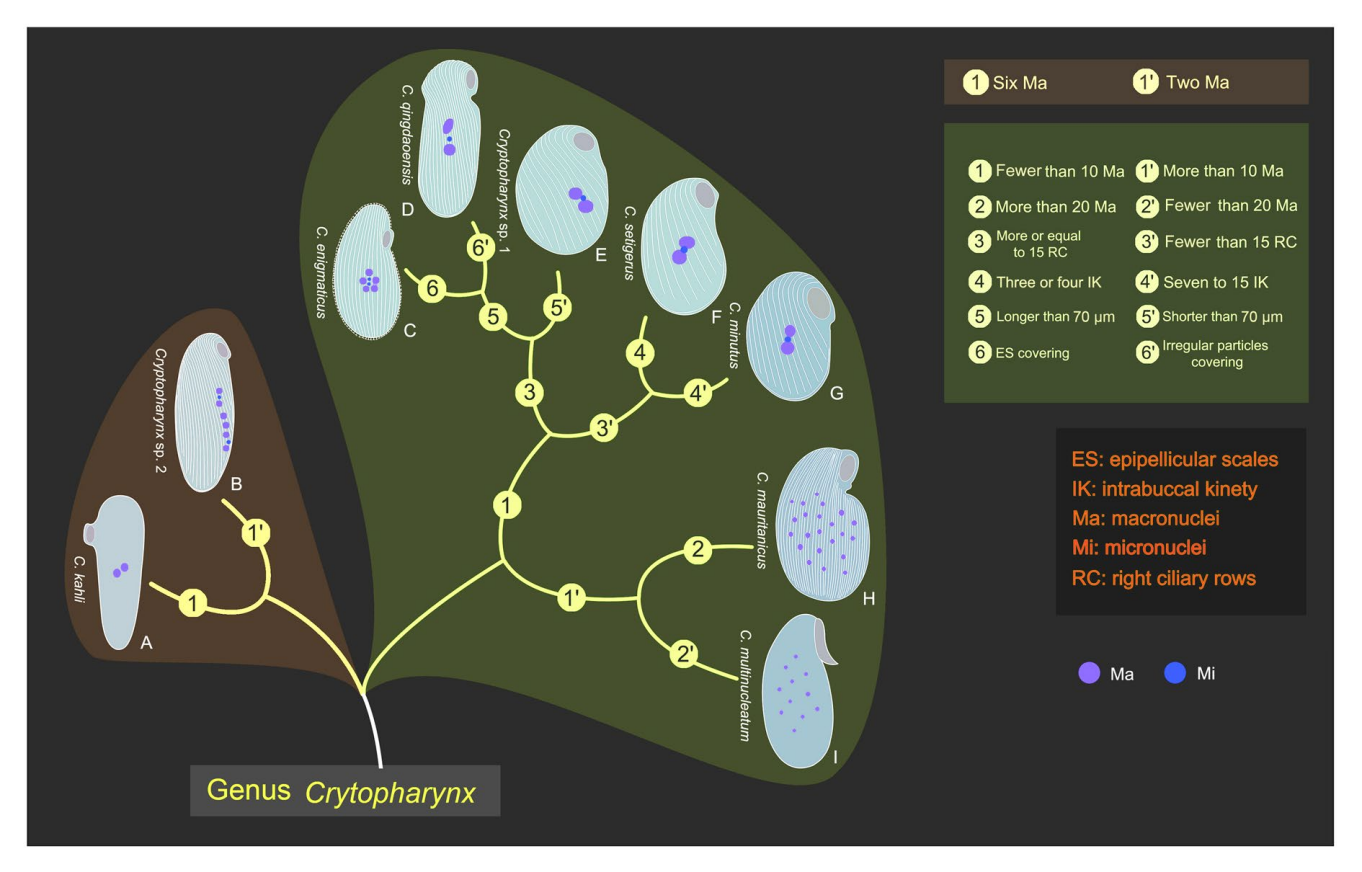
Illustrated key to valid *Cryptopharynx* species. **A** *Cryptopharynx kahli,* redrawn from Carey (1992). **B** *Cryptopharynx* sp. 2, redrawn from Foissner (1995). **C** *Cryptopharynx enigmaticus*, redrawn from Dragesco (1960). **D** *Cryptopharynx qingdaoensis* n. sp. **E** *Cryptopharynx* sp. 1, redrawn from Foissner (1995). **F** *Cryptopharynx setigerus,* redrawn from Foissner (1995). **G** *Cryptopharynx minutus*, redrawn from Xu et al. (2011a). **H** *Cryptopharynx mauritanicus*, redrawn from Dragesco (1965). **I** *Cryptopharynx multinucleatum,* redrawn from Dragesco (1960). Scale bar: 70 µm (**A**, **B**); 50 µm (**C**, **D**, **H**, **I**); 25 µm (**E**, **F**, **G**)

1 Cell length in vivo longer than 110 µm………………2

1.1 Cell length in vivo shorter than 110 µm………………3

2 Six macronuclei forming chain in central cell………………………………………………*Cryptopharynx* sp. 2

2.1 Two macronuclei located in center of cell…………………………………………*Cryptopharynx kahli*

3 More than 10 macronuclei, scattered in cell………4

3.1 Fewer than 10 macronuclei, located in center of cell………5

4 More than 20 macronuclei………………………*Cryptopharynx mauritanicus*

4.1 Fewer than 20 macronuclei…………………*Cryptopharynx multinucleatum*

5 Fewer than 15 right lateral ciliary rows……………6

5.1 More than 15 right lateral ciliary rows……………7

6 3–4 dikinetids in intrabuccal kinety……………………………*Cryptopharynx setigerus*

6.1 7–15 dikinetids in intrabuccal kinety…………………………………………………….*Cryptopharynx minutus*

7 Cell length in vivo longer than 70 µm……………………8

7.1 Cell length in vivo shorter than 70 µm…………………*Cryptopharynx* sp. 1

8 Epipellicular scales covering left side……………………………*Cryptopharynx enigmaticus*

8.1 Irregular particles covering left side……………………………*Cryptopharynx qingdaoensis*

### Revision of the genus *Cryptopharynx* Kahl, 1928

*Improved diagnosis.* Cell flat, oval in outline, with elliptical protruding oral area. Left cell surface covered with scales or irregular particles embedded in mucous layer. Buccal kineties continuous, intrabuccal kinety short and composed of few kinetids forming a slightly curved row. Marine interstitial. Type species: *Cryptopharynx setigerus* Kahl, 1928.

### ***Cryptopharynx setigerus ***Kahl, 1928 (Fig. 11F)

1928 *Cryptopharynx setigerus* Kahl, Arch Hydrob, 19: 81–84, Fig. 17(a–e).

1934 *Cryptopharynx setigerus* Kirby, Archiv Protistenknd, 82: 119–121, Fig. 10–15, 17.

1960 *Cryptopharynx setigrum* Dragesco, Trav Stat Biol, 12: 256 (no illustration).

1965 *Cryptopharynx setigerus* Dragesco, Cah Biol Mar, 6: 389 (no illustration).

1992 *Cryptopharynx setigerus* Carey, Marine Interstitial Ciliates: An Illustrated Key, 101, Fig. 347.

1996 *Cryptopharynx setigerus* Foissner, Archiv Protistenknd, 146: 309–327, Fig. 1–13.

*Cryptopharynx setigerus* was first recorded by Kahl (1928) with a brief description based on live characters and stained nuclei, and was redescribed by Kirby (1934). Dragesco (1960, 1965) isolated it in Roscoff (France) and Mauritania (Africa) with brief descriptions without illustrations. Carey (1992) redefined this species*.* Although *C. setigerus* has been known for a long time, its oral structure was not reported until 1996 (Foissner 1996).

*Diagnosis.* Cell length about 25–96 µm in vivo. Cell leaf-like with a hump rising from the left surface. Elliptical concave buccal area. Left lateral surface covered with mucous layer. Six to 18 right ciliary rows. First and second right buccal kinety and intrabuccal kinety composed of 14–26, 5–14 and 3–4 dikinetids, respectively. Nuclear apparatus near center of cell, consisting of two or three macronuclei and one micronucleus.

*Habitat.* Marine and brackish water interstitial habitats.

*Distribution.* Found in brackish water in Bad Oldesloe, Germany (Kahl 1928) and Elkhorn Slough, California, USA (Kirby 1934). Found in seawater in Roscoff, France (Foissner 1995) and on Romanian and Bulgarian coasts of the Black Sea (Azovsky and Mazei 2003a; Kovaleva and Golemansky 1979).

*Gene sequences.* Unavailable.

### ***Cryptopharynx kahli*** Dragesco, 1954 (Fig. 11A)

1954 *Cryptopharynx kahli* Dragesco, Bull Soc Zool, 1: 65, Fig. 1(j).

1960 *Cryptopharynx setigerum* var. *furcatum* Dragesco, Trav Stat Biol, 12: 255, Fig. 131a.

1992 *Cryptopharynx kahli* Carey, Marine Interstitial Ciliates: An Illustrated Key, 101, Fig. 350.

*Cryptopharynx kahli* was first reported from Roscoff by Dragesco (1954a, b) with simple figures and a brief description based on observations of specimens in vivo. *Cryptopharynx setigerum* var. *furcatum* was also found in Roscoff and differs from the type species *Cryptopharynx setigerum* in a number of features, including its larger size (cell length in vivo 130 µm) and flattened body that is pointed posteriorly, which are consistent with the morphology of *Cryptopharynx kahli* (Dragesco 1960). Carey (1992) redefined this species listing *C. setigerum* var. *furcatum* as a junior synonym of *C. kahli.* There have been no further redescriptions, therefore the following diagnosis is based on Dragesco (1954a, b, 1960).

*Diagnosis.* Cell size around 130–135 µm in vivo, leaf-like with a hump rising from the left surface, a flat right side, and a protruding oral structure. Nuclear apparatus comprising two macronuclei. Pigments brown.

*Habitat.* Marine interstitial.

*Distribution.* Found in sand at Roscoff (France) (Dragesco 1954a, b).

*Gene sequences.* Absent.

### ***Cryptopharynx enigmaticus ***Dragesco, 1954 (Fig. 11C)

1954 *Cryptopharynx enigmaticus* Dragesco, Bull Soc Zool, 1: 65, Fig. 2h.

1960 *Cryptopharynx enigmaticus* Dragesco, Trav Stat Biol, 12: 257–258, Fig. 132(a–e).

1992 *Cryptopharynx enigmaticus* Carey, Marine Interstitial Ciliates: An Illustrated Key, 101, Fig. 345.

*Cryptopharynx enigmaticus* was first isolated from Roscoff, France, with simple illustrations and a brief description based on observations of living cells (Dragesco 1954a, b). It was later reported from the same locality with more detailed illustrations and a more detailed description (Dragesco 1960). Carey (1992) redefined this species*.* Based on this information, the diagnosis is as follows.

*Diagnosis.* Cells medium size in vivo 70–100 µm, flattened and asymmetrical. Right side flat, left side bulged with pointed, button-like scales. 17 right ciliary rows. Nuclear apparatus consists of 1–5 macronuclei and on average three micronuclei.

*Habitat.* Marine interstitial.

*Distribution.* Found in sand at Roscoff, France (Dragesco 1954a, b, 1960).

*Gene sequences.* Unavailable.

### ***Cryptopharynx multinucleatum ***Dragesco, 1960 (Fig. 11I)

1954 *Cryptopharynx multinucleatum* Dragesco, Bull Soc Zool, 1: 65, Fig. 1(i).

1960 *Cryptopharynx multinucleatum* Dragesco, Trav Stat Biol, 12: 256, Fig. 131b.

1992 *Cryptopharynx multinucleatum* Carey, Marine Interstitial Ciliates: An Illustrated Key, 101, Fig. 348.

*Cryptopharynx multinucleatum* was first reported by Dragesco (1954a, b) with schematic figures and a brief morphological description of living cells. A more detailed description with more detailed illustrations were added shortly thereafter (Dragesco 1960). Carey (1992) redefined this species*.* Based on this information, the diagnosis is as follows.

Diagnosis. Cell in vivo about 90 µm with a large oral structure. Nuclear apparatus comprising 10–14 macronuclei and numerous micronuclei. Pigment granules distributed around oral structure.

*Habitat.* Marine interstitial.

*Distribution.* Found in sand of Verte island channel at Roscoff, France (Dragesco 1954a, b, 1960).

*Gene sequences.* Unavailable.

### ***Cryptopharynx mauritanicus ***Dragesco, 1965 (Fig. 11H)

1965 *Cryptopharynx mauritanicus* Dragesco, Cah Biol Mar, 6: 387–388, Fig. 23.

1992 *Cryptopharynx mauritanicus* Carey, Marine Interstitial Ciliates: An Illustrated Key, 101, Fig. 352.

*Cryptopharynx mauritanicus* was first isolated from Mauritania with schematic figures and a brief description of living cells (Dragesco 1965). Carey (1992) redefined this species*.* Based on this information, the diagnosis is as follows.

*Diagnosis*. Cells medium size in vivo, around 110 µm long, flattened and asymmetrical with right side flat and left side bulged. More than 40 right ciliary rows. Somatic cilia about 8 µm in length. Nuclear apparatus consisting of 50–70 macronuclei described as “pulverized” spheroidal elements; micronuclei not observed. Sand grains attached to mucus layer on left side of cell. Right side, and to a lesser extent left side, with protrusions of the pellicle.

*Habitat.* Marine interstitial.

*Distribution.* Found in sand of beaches at Port Étienne, Mauritania (Dragesco 1965).

*Gene sequences.* Unavailable.

### *Cryptopharynx* sp. 1 (Fig. 11E)

1996 *Cryptopharynx* sp. 1 Foissner, Archiv Protistenknd, 146: 315, Figs. 14–15.

*Cryptopharynx* sp. 1 was reported by Foissner (1995) based on a few specimens mixed with those of *Cryptopharynx setigerus* from Roscoff, France. These two species are distinguished by differences in their ciliature. However, *Cryptopharynx* sp. 1 lacks morphological characteristics in vivo, therefore it has not been formally described or named.

*Diagnosis.* Cell size around 50 µm after protargol staining. About 15 right ciliary rows. Intrabuccal kinety composed of ca. 8 dikinetids. Nuclear apparatus near center of cell consisting of two macronuclei and one micronucleus.

*Habitat.* Marine interstitial.

*Distribution.* Found in sand at Roscoff, France (Foissner 1995).

*Gene sequences.* Unavailable.

### *Cryptopharynx* sp. 2 (Fig. 11B)

1996 *Cryptopharynx* sp. 2 Foissner, Arch Protistenkd, 146: 315, Fig. 16–20.

*Cryptopharynx* sp. 2 was reported by Foissner (1995) based on two specimens mixed with those of *Cryptopharynx setigerus* from Roscoff, France. These two species are distinguished by differences in their ciliature. However, *Cryptopharynx* sp. 2 lacks observations based on live cells, therefore it has not been formally described or named.

*Diagnosis.* Cell size around 130 µm after protargol staining. Right side flat and left side bulged with furcated scales. About 22 right ciliary rows (from illustration). Intrabuccal kinety composed of ca. nine dikinetids (from illustration). Nuclear apparatus near center of cell consisting of six macronuclei and two micronuclei (from illustration).

*Habitat.* Marine interstitial.

*Distribution.* Found in sand at Roscoff, France (Foissner 1995).

*Gene sequences.* Unavailable.

### ***Cryptopharynx minutus*** Xu et al., 2017 (Fig. 11G)

2017 *Cryptopharynx minutus* Xu et al., Eur J Protistol, 58: 77–86, Fig. 4–5.

*Cryptopharynx minutus* was reported by Xu et al. (2017) who described this species in detail using modern methods, with the following diagnosis.

*Diagnosis*. Size in vivo about 40 × 20 µm, ovoidal with elliptical protruding oral area. Left lateral surface covered by epipellicular scales. 10–14 right ciliary rows. First and second right buccal kinety and intrabuccal kinety composed of 47–55, 37–45 and 7–15 dikinetids, respectively. Nuclear apparatus near center of cell, consisting of two to five macronuclei and one or two micronuclei.

*Habitat.* Marine interstitial.

*Distribution.* Found in seawater at Qingdao, China (Xu et al. 2017).

*Gene sequences.* Unavailable.

### ***Cryptopharynx qingdaoensis*** n. sp. (Fig. 11D)

*Diagnosis.* See above.

*Type locality and distribution.* See above.

*Gene sequences.* See above.

### Genus ***Kentrophoros*** Sauerbrey, 1928

The genus *Kentrophoros* was established by Sauerbrey (1928). *Centrophorus* Kahl, 1928, *Centrophorella* Kahl, 1935 and *Centrophoros* Müller & Henle, 1937 are all synonyms of *Kentrophoros*. The variations of any given species name are provided below for ease of access to historic texts. *Kentrophorus* is a unique psammophilic ciliate genus in that it carries a symbiotic “kitchen garden” of sulphur bacteria on its left side providing the bulk of its nutrients (Fenchel and Finlay 1989), the oral apparatus being either vestigial or absent (Foissner 1995). Foissner (1995) provided a list of the 12 valid species of *Kentrophoros.* Currently there are still 12 nominal species assigned to this genus (see Discussion). Among these, details of the ciliature are known for only three, namely *K. fistulosus*, *K. flavus* and *K. gracilis*. With the addition of new data from the present study, four sequences belonging to these three species are now available. Furthermore, there are many unidentified sequences the National Center for Biotechnology Information database that lack morphological data or vouchered specimensin. Here we provide a key to the identification of the 12 valid *Kentrophoros* species and provide a generic revision.

### Key to valid species of *Kentrophoros* (Fig. 12)

**Fig. 12:**
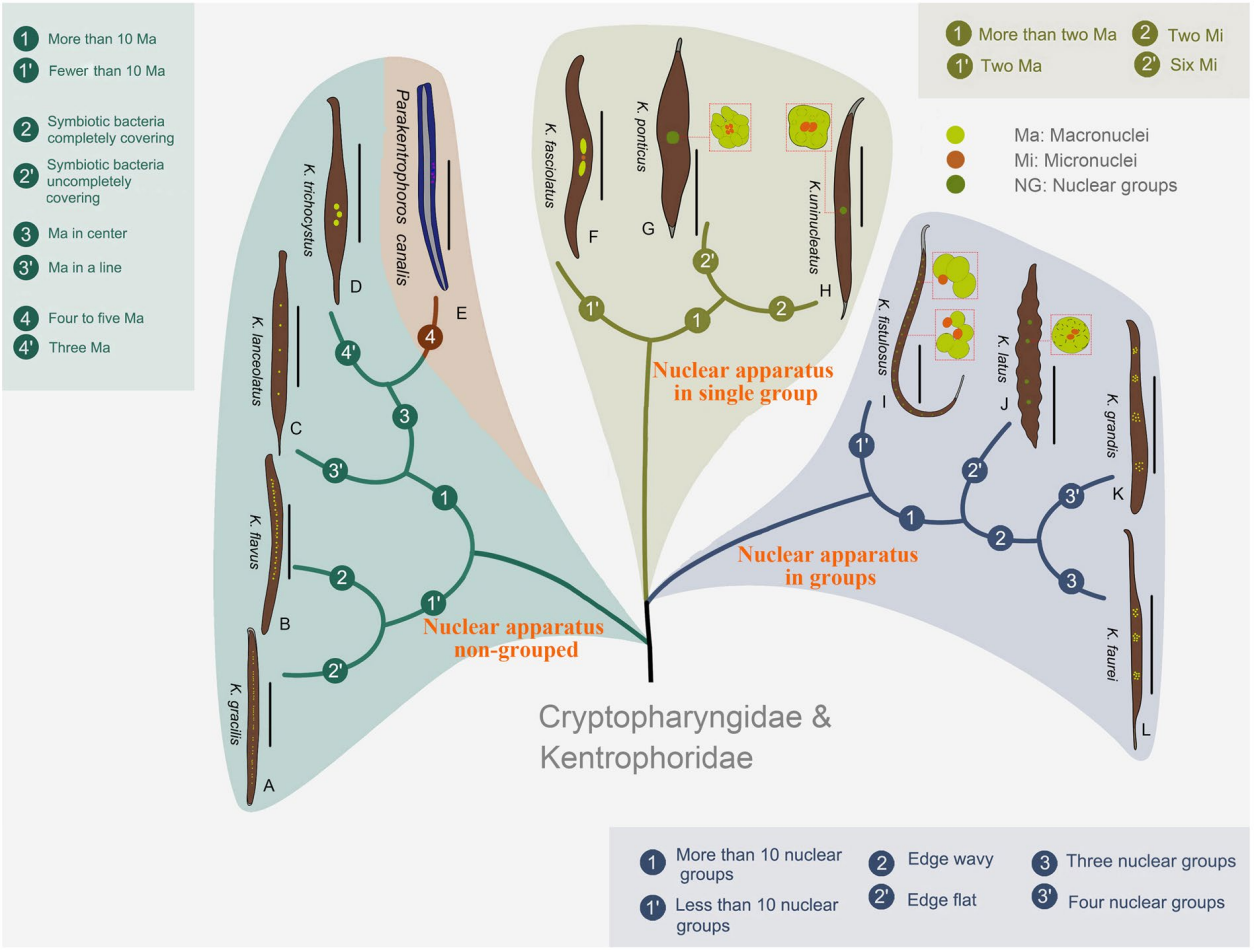
Illustrated key to valid *Kentrophoros* and *Parakentrophoros* species*.* **A** *Kentrophoros gracilis,* redrawn from Xu et al. (2011a). **B** *Kentrophoros flavus,* redrawn from Xu et al. (2011a). **C** *Kentrophoros lanceolatus*, redrawn from Fauré-Fremiet (1951). **D** *Kentrophoros trichocystus*, redrawn from Dragesco (1954a, b). **E** *Parakentrophoros canalis.* **F** *Kentrophoros fasciolatus*, redrawn from Sauerbrey (1928). **G** *Kentrophoros ponticus*, redrawn from Kovaleva (1966). **H** *Kentrophoros uninucleatus*, redrawn from Raikov (1962). **I** *Kentrophoros fistulosus*, redrawn from Foissner (1995). **J** *Kentrophoros latus*, redrawn from Raikov (1962). **K** *Kentrophoros grandis*, redrawn from Dragesco (1954a, b). **L** *Kentrophoros faurei*, redrawn from Dragesco (1954a, b). Scale bar: 300 µm (**E**, **F**, **J**–*L*); 200 µm (**A**–**C**, **G**, **H**); 100 µm (**D**, **I**)

1 Ovoid or pyriform cell shape…………………………………………………………………….*Kentrophoros minutus*

1.1 Cell flattened and ribbon-shaped……………2

2 Nuclear apparatus not grouped…………………3

2.1 Nuclear apparatus in group/groups……………4

3 Fewer than 10 macronuclei………………5

3.1 More than 10 macronuclei………………6

4 Nuclei in a single group……………………7

4.1 Nuclei in two or more groups………………8

5 Macronuclei located in central region of cell………………………………………..*Kentrophoros trichocystus*

5.1 Macronuclei in a line…………………………………………………*Kentrophoros lanceolatus*

6 Symbiotic bacteria completely covering the left side……………………………*Kentrophoros flavus*

6.1 Symbiotic bacteria incompletely covering the left side……………………………..*Kentrophoros gracilis*

7 More than two macronuclei………………………9

7.1 Two macronuclei……………………………………………………*Kentrophoros fasciolatus*

8 Fewer than 10 nuclear groups…………………………10

8.1 More than 10 nuclear groups……………………………………………*Kentrophoros fistulosus*

9 Two micronuclei…………………………………………………*Kentrophoros uninucleatus*

9.1 Six micronuclei……………………………………………………………………………………*Kentrophoros ponticus*

10 Cell margin wavy…………………………………………………………………*Kentrophoros latus*

10.1 Cell margin not wavy…………………………………11

11 Three nuclear groups…………………………………………………………*Kentrophoros faurei*

11.1 Four nuclear groups…………………………………………………………*Kentrophoros grandis*

### Revision of the genus *Kentrophoros* Sauerbrey, 1928

*Improved diagnosis.* Cell flattened, often C-shaped in cross-section. Right side densely ciliated, covered by ciliated dikinetids arranged in kineties; left side covered by mucous material colonized by symbiotic sulphur bacteria, with a “bristle” kinety along the cell margin. Oral region highly reduced to remnants of kinetids. Nuclei in clusters. Marine interstitial. Type species: *Kentrophoros fasciolatus.*

### ***Kentrophoros fasciolatus ***(Sauerbrey, 1928) Foissner, 1995 (Fig. 12F)

1928 *Kentrophoros fasciolatum* Sauerbrey, Arch Protistenkd, 62: 381–382, Fig. 45–46.

1933 *Centrophorella fasciolata* Kahl, Lepzig: Akademische Verlagsgesellschaft Becker & Erler, 74, Fig. 5 (32).

1935 *Centrophorella fasciolata* Kahl, Tierwelt Dtl, 30: 826, Fig. 150 (42).

1937 *Kentrophoros fasciolatum* Noland, Trans Amer Micros Soc, 56: 163, Fig. 2 (A–C).

1950 *Centrophorella fasciolata* Fauré-Fremiet, Bull Biol Fr Belg, 84: 54–56, Fig. 10–11.

1960 *Centrophorella fasciolata* Dragesco, Trav Stat Biol, 12: 177, Fig. 80 (g), 81.

1965 *Kentrophoros fasciolatus* Dragesco, Cah Biol Mar, 6: 381 (no illustration).

1992 *Kentrophoros fasciolatum* Carey, Marine Interstitial Ciliates: An Illustrated Key, 89, Fig. 291.

1995 *Kentrophoros fasciolatus* Foissner, Archiv Protistenknd, 146: 166 (no illustration).

*Kentrophoros fasciolatus* was first reported from Kieler Förde, Germany, under the name *Kentrophoros fasciolatum,* with a description based on observations o live specimens (Sauerbrey 1928). It was subsequently isolated from Kieler Förde (Germany), Helgoland (Germany) and Florida (USA) (Kahl 1933, 1935; Noland 1937), each time under a different name (see list above). Based on a population from France, Fauré-Fremiet (1950) provided detailed observations of live and fixed specimens showing the nuclear group. Subsequently, Dragesco (1960) redescribed this species based on populations isolated from France where it was found in great abundance. Carey (1992) redefined this species*.* Foissner (1995) revised this genus and confirmed the species name *Kentrophoros fasciolatus.*

*Diagnosis.* Cell length 100–1600 µm in vivo, flattened and ribbon-like. About 6–7 right ciliary rows. One nuclear group near center of cell consisting of two macronuclei and one micronucleus. One side is slightly yellow. Densely arranged epibiontic bacteria covering left side of cell.

*Habitat.* Marine interstitial.

*Distribution.* Found in sandy coastal basins and beach Kieler Förde, Germany (Sauerbrey 1928; Kahl 1933, 1935). Found in sand at Helgoland (Germany) (Kahl 1933, 1935). Found in sea water in sediment over a sandy bottom in Florida, USA (Noland 1937). Found in sand at Roscoff and Banyuls-sur-Mer (France) (Fauré-Fremiet 1950). Found in sea waters of Romanian, Bulgarian and Northeastern (from Adler to Anapa) parts of the Black Sea coasts (Azovsky and Mazei 2003; Kovaleva and Golemansky 1979).

*Gene sequences.* Unavailable.

### ***Kentrophoros fistulosus ***(Fauré-Fremiet, 1950) Foissner, 1995 (Fig. 12I)

*Diagnosis.* See above.

*Type locality and distribution.* See above.

*Gene sequences.* See above.

### ***Kentrophoros lanceolatus ***(Fauré-Fremiet, 1951) Foissner, 1995 (Fig. 12C)

1951 *Centrophorella lanceolata* Fauré-Fremiet, Biol Bull Mar Biol Lab, 100: 65–67, Fig. 2.

1960 *Centrophorella lanceolata* Dragesco, Trav Stat Biol, 12: 179, Fig. 80 (e)*.*

1992 *Kentrophoros lanceolata* Carey, Marine Interstitial Ciliates: An Illustrated Key, 90, Fig. 295.

1995 *Kentrophoros lanceolatus* Foissner, Archiv Protistenknd, 146: 166 (no illustration).

*Kentrophoros lanceolatus* was first isolated Cape Cod (Massachusetts, United States) and described under the name *Centrophorella lanceolata,* based on observations of both live and stained specimens (Fauré-Fremiet 1951). It was later isolated at Roscoff, France, under the same name (Dragesco 1960). Carey (1992) redefined this species and transferred it to *Kentrophorus* as *Kentrophoros lanceolata.* Foissner (1995) renamed this species as *Kentrophoros lanceolatus*.

*Diagnosis.* Cell 460–520 µm long in vivo, flattened, ribbon-like, and narrowed at both ends. Five or six macronuclei arranged along cell meridian without forming nuclear groups. Bacterial symbionts colorless.

*Habitat.* Marine interstitial.

*Distribution.* Found in sand at Cape Cod, Massachusetts, USA (Fauré-Fremiet 1951) and at Roscoff, France (Dragesco 1960).

*Gene sequences.* Unavailable.

### ***Kentrophoros faurei ***(Dragesco, 1954) Foissner, 1995 (Fig. 12L)

1954 *Centrophorella faurei* Dragesco, Bull Soc Zool, 1: 61, Fig. 2(f).

1960 *Centrophorella faurei* Dragesco, Trav Stat Biol, 12: 181, Fig. 83.

1992 *Kentrophoros faurei* Carey, Marine Interstitial Ciliates: An Illustrated Key, 89, Fig. 288.

1995 *Kentrophoros faurei* Foissner, Archiv Protistenknd, 146: 166 (no illustration).

*Kentrophoros faurei* was first reported under the name *Centrophorella faurei* by Dragesco (1954a, b), based on observations of live specimens from Roscoff, France, and later from the same locality (Dragesco 1960). Carey (1992) redefined this species and transferred it to *Kentrophorus* as *Kentrophoros faurei*, but mistakenly gave the authority as Dragesco, 1953 instead of Dragesco 1954a, b. Foissner (1995) revised this genus and confirmed the species name *Kentrophoros faurei.*

*Diagnosis.* Cell 750–1000 µm long in vivo, flattened and ribbon-like with a well-developed neck and pointed tail. Dense bacterial assemblage rendering cell almost entirely black. Three nuclear groups, each one consisting of 20–25 macronuclei and approximately six micronuclei.

*Habitat.* Marine interstitial.

*Distribution.* Found in sand in Roscoff and Banyuls-sur-Mer, France (Dragesco 1954a, b, 1960).

*Gene sequences.* Unavailable.

### ***Kentrophoros grandis ***(Dragesco, 1954) Foissner, 1995 (Fig. 12K)

1954 *Centrophorella grandis* Dragesco, Bull Soc Zool, 1: 61, Fig. 2e.

1960 *Centrophorella grandis* Dragesco, Trav Stat Biol, 12: 180, Fig. 82.

1992 *Kentrophoros grandis* Carey, Marine Interstitial Ciliates: An Illustrated Key, 90, Fig. 294.

1995 *Kentrophoros grandis* Foissner, Archiv Protistenknd, 146: 166 (no illustration).

*Kentrophoros grandis* was first reported from Roscoff (France) under the name *Centrophorella grandis* with a description based on observations of live specimens (Dragesco 1954a, b). Carey (1992) redefined this species and transferred it to *Kentrophoros* as *Kentrophoros grandis.* Foissner (1995) accepted the validity of this species without a detailed report.

*Diagnosis.* Cell flattened, length in vivo exceeding 1000 µm, width 110 µm. The nuclear apparatus is made up of 4–6 groups of 5–8 macronuclei, yielding a total of 27–30 macronuclei, and 7–9 micronuclei. Bacterial symbionts colorless and large, giving the cell an almost black appearance.

*Habitat.* Marine interstitial.

*Distribution.* Found in sand at Roscoff, France (Dragesco 1954a, b).

*Gene sequences.* Unavailable.

### ***Kentrophoros trichocystus ***(Dragesco, 1954) Foissner, 1995 (Fig. 12D)

1954 *Centrophorella trichocystus* Dragesco, Bull Soc Zool, 1: 62, Fig. 2g.

1960 *Centrophorella trichocystus* Dragesco, Trav Stat Biol, 12: 180, Fig. 84.

1992 *Kentrophoros trichocystus* Carey, Marine Interstitial Ciliates: An Illustrated Key, 90. Figure 301.

1995 *Kentrophoros trichocystus* Foissner, Archiv Protistenknd, 146: 167 (no illustration).

*Kentrophoros trichocystus* was first reported from Roscoff (France) under the name *Centrophorella trichocystus* and was described based on observations of live specimens (Dragesco 1954a, b). It was later reported from the same locality (Dragesco 1960). Carey (1992) made a simple definition of this species and transferred it to *Kentrophorus* as *Kentrophoros trichocystus*. Foissner (1995) included this in a list of valid species of *Kentrophoros* without giving further details. The infraciliature of this species remains unknown.

*Diagnosis.* Cell 250 µm long in vivo, lanceolate with distinctly widened cell body and narrowed neck and tail, and transparent (with a light-yellow hue). Anterior region curved, tail rounded. Three distinct macronuclei located in mid-region of cell. Large trichocysts present. Symbiotic bacteria covering entire left side.

*Habitat.* Marine interstitial.

*Distribution.* Found in sand at Roscoff, France (Dragesco 1954a, b).

*Gene sequences.* Unavailable.

### ***Kentrophoros minutus ***(Dragesco, 1960) Foissner, 1995

1960 *Centrophorella minuta* Dragesco, Trav Stat Biol, 12: 184 (no illustration).

1992 *Kentrophoros minuta* Carey, Marine Interstitial Ciliates: An Illustrated Key, 90 (no illustration).

1995 *Kentrophoros minutus* Foissner, Archiv Protistenknd, 146: 166 (no illustration).

*Kentrophoros minutus* was first reported from Roscoff (France) under the name *Centrophorella minuta* and was described based on observations of live specimens (Dragesco 1960). Carey (1992) redefined this species and transferred it to *Kentrophorus* as *Kentrophoros minuta*. Foissner (1995) revised this species as *Kentrophoros minutus* without giving further details. Illustrations of this species are lacking.

*Diagnosis.* Cell 170 µm long in vivo, oval or pyriform with a distinctly curved “beak” region and a rounded posterior. Symbiotic bacteria present on the left surface. About 10 right somatic kineties.

*Habitat.* Marine interstitial.

*Distribution.* Found in sand at Roscoff, France (Dragesco 1960).

*Gene sequences.* Unavailable.

### ***Kentrophoros latus ***(Raikov, 1962) Foissner, 1995 (Fig. 12J)

1962 *Kentrophoros latum* Raikov, Cah Biol Mar, 3: 354–356. Figure 12

1992 *Kentrophoros latum* Carey, Marine Interstitial Ciliates: An Illustrated Key, 90, Fig. 296.

1995 *Kentrophoros latus* Foissner, Archiv Protistenknd, 146: 166 (no illustration).

*Kentrophoros latus* was first reported from the White Sea in 1962 under the name *Kentrophoros latum,* with a description based on observations of both live and stained specimens (Raikov 1962)*.* Carey (1992) redefined this species*.* Foissner (1995) renamed this species as *Kentrophoros latus* without further details.

*Diagnosis.* Cell 600–1200 µm long in vivo, ribbon-like, with undulating lateral margins. Distinct head region and pointed tail. 30–45 kineties. 1–4 nuclear groups, each consisting of four macronuclei and 1–4 (usually two) micronuclei.

*Habitat.* Marine interstitial.

*Distribution.* Found in Barents Sea and White Sea (Raikov 1962).

*Gene sequences.* Unavailable.

### ***Kentrophoros uninucleatus ***(Raikov, 1962) Foissner, 1995 (Fig. 12H)

**Fig. 13:**
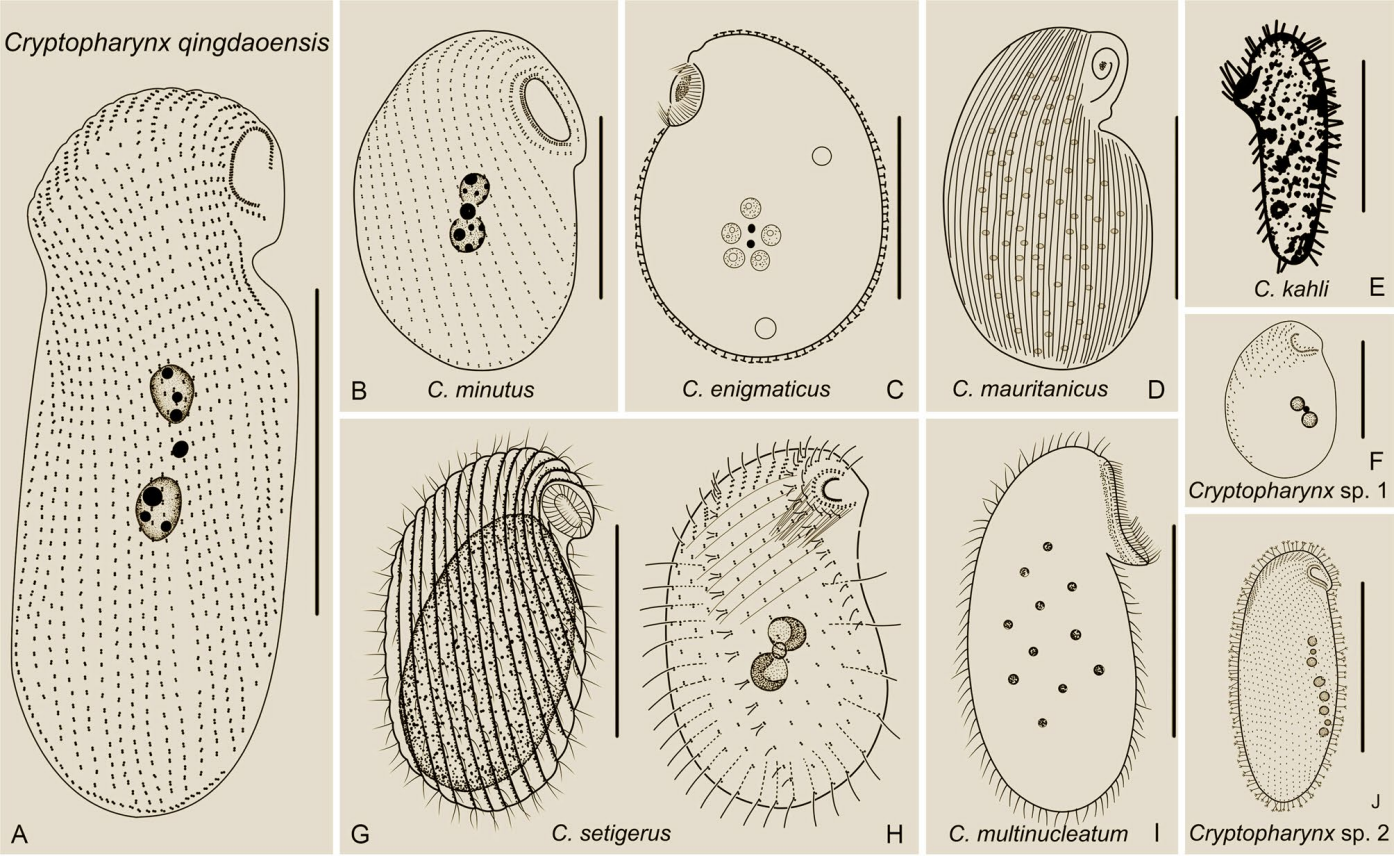
Illustrations of *Cryptopharynx* species. **A** *Cryptopharynx qingdaoensis* n. sp. **B** *Cryptopharynx minutus*, redrawn from Xu et al. (2017). **C** *Cryptopharynx enigmaticus*, redrawn from Dragesco (1960). **D** *Cryptopharynx mauritanicus*, redrawn from Dragesco (1965). **E** *Cryptopharynx kahli*, redrawn from Dragesco (1954a, b). **F** *Cryptopharynx* sp. 1, redrawn from Foissner (1995). **G**, **H** *Cryptopharynx setigerus*, redrawn from Foissner (1995). **I** *Cryptopharynx multinucleatum*, redrawn from Dragesco (1960). **J** *Cryptopharynx* sp. 2, redrawn from Foissner (1995). Scale bars: 60 µm (**E**, **J**); 50 µm (**A**, **C**, **D**, **I**); 25 µm (**B**, **F**, **G**, **H**)

1962 *Kentrophoros uninucleatum* Raikov, Cah Biol Mar, 3: 356–357. Figure 13.

1992 *Kentrophoros uninucleatum* Carey, Marine Interstitial Ciliates: An Illustrated Key, 91. Figure 299.

1995 *Kentrophoros uninucleatus* Foissner, Archiv Protistenknd, 146: 167 (no illustration).

*Kentrophoros uninucleatus* was first reported from the White Sea under the name *Kentrophoros uninucleatum* with a brief description based on observations of live specimens (Raikov 1962). Carey (1992) made a simple definition of this species and Foissner (1995) renamed it *Kentrophoros uninucleatus*. The infraciliature of this species remains unknown.

*Diagnosis.* Cell 550–650 µm long in vivo, flattened with pointed head and tail. 16–17 right somatic kineties. Single nuclear group in central region of cell containing numerous macronuclei and two distinct micronuclei.

*Habitat.* Marine interstitial.

*Distribution.* Found in White Sea (Raikov 1962).

*Gene sequences.* Unavailable.

### ***Kentrophoros gracilis ***(Raikov, 1963) Foissner, 1995 (Fig. 12A)

*Diagnosis.* See above.

*Type locality and distribution.* See above.

*Gene sequence*: See above.

### ***Kentrophoros ponticus ***(Kovaleva, 1966) Foissner, 1995 (Fig. 12G)

1966 *Kentrophoros ponticum* Kovaleva, Zool Zh, 45: 1608–1609. Figure 7.

1992 *Kentrophoros ponticum* Carey, Marine Interstitial Ciliates: An Illustrated Key, 90. Figure 293.

1995 *Kentrophoros ponticus* Foissner, Archiv Protistenknd, 146: 167 (no illustration).

*Kentrophoros ponticus* was first reported from the Black Sea under the name *Kentrophoros ponticum* with a description based on observations of live specimens (Kovaleva 1966). Carey (1992) made a simple definition of this species and Foissner (1995) renamed it *Kentrophoros ponticus* without further details. The infraciliature of this species remains unknown.

*Diagnosis.* Cell 300–400 µm long in vivo, flattened with pointed anterior and posterior. 17–20 right somatic kineties. Single nuclear group consisting of 6–12 macronuclei and six micronuclei. Symbiotic bacteria covering most of left side except anterior and posterior regions.

*Habitat.* Marine interstitial.

*Distribution.* Found at Crimean, Bulgarian and Notheastern parts of the Black Sea coasts (Azovsky and Mazei 2003; Kovaleva 1966; Kovaleva and Golemansky 1979).

*Gene sequences.* Unavailable.

### ***Kentrophoros flavus ***(Raikov & Kovaleva, 1968) Foissner, 1995 (Fig. 12B)

1968 *Kentrophoros flavum* Raikov and Kovaleva, Acta Protozool, 6: 325–327. Figure 11

1992 *Kentrophoros flavum* Carey, Marine Interstitial Ciliates: An Illustrated Key, 89, Fig. 292.

1995 *Kentrophoros flavus* Foissner, Archiv Protistenknd, 146: 166 (no illustration).

2011 *Kentrophoros flavus* Xu et al., Eur J Protistol, 47: 179–182. Figure 4–5.

*Kentrophoros flavus* was first reported from Ussuri Bay (Russia) under the name *Kentrophoros flavum* with a description based on observations of both live and Feulgen-stained specimens (Raikov and Kovaleva 1968). Carey (1992) redefined this species. Foissner (1995) reorganized this genus and renamed this species *Kentrophoros flavus.* Xu et al. (2011a) provided morphometric data and details of the infraciliature based on observations of a Chinese population.

*Diagnosis.* Cell about 250–600 µm long in vivo and highly flattened. 9–49 macronuclei and 3–17 micronuclei arranged in a long irregular row. About 12–19 somatic kineties on right side of cell. Densely packed epibiontic bacteria covering left side of cell.

*Habitat.* Marine interstitial.

*Distribution.* Found in sand at Ussuri Bay, Russia, Bulgarian coast of the Black Sea, and at Qingdao, China (Azovsky and Mazei 2003; Kovaleva and Golemansky 1979; Raikov and Kovaleva 1968; Xu et al. 2011a).

*Gene sequences.* Unavailable.

### Phylogenetic analyses

Several phylogenetic analyses have been conducted to illustrate the evolutionary relationships among the Karyorelictea (Ma et al. 2022a, b; Xu et al. 2013; Yan et al. 2016). It was recently shown that Protostomatida is monophyletic and groups with Loxodida far away from Wilbertomorphida and Protoheterotrichida Nouzarède, 1977 (Ma et al. 2022a). In previous studies, Kentrophoridae and Cryptopharyngidae belonged to Protostomatida and Loxodida, respectively, although the cells of both Kentrophoridae and Cryptopharyngidae are flattened (Lynn 2008). Since Kentrophoridae and Cryptopharyngidae are difficult to collect and stain for identification, their evolutionary relationships are poorly studied.

In recent years, there have been some attempts to reveal the relationships of karyorelicteans according to their oral structure (Foissner 1995, 1996, 1998; Foissner and Al-Rasheid 1999). Ma et al. (2022a) combined morphology and molecular phylogeny to analyse the relationships within Karyorelictea. Geleiidae can be distinctly separated from other taxa within Karyorelictea by having a completely ciliated cell and a complex oral structure (Méndez-Sánchez et al. 2022). Wilbertomorphidae branches early within the Karyorelictea. Nevertheless, based on the reniform and bilaterally flattened body, highly reduced cytostome positioned subapically on the narrow ventral surface, dikinetid somatic kineties on the right surface, and a single kinety encircling the margin on the left side, Wilbertomorphidae may have a close relationship with Loxodidae (Ma et al. 2022a, b; Xu et al. 2013). Kentrophoridae and Trachelocercidae belong to the order Protostomatida due to the slender and highly contractile cell, right side densely ciliated, left side with a glabrous area of varying width bordered by a “bristle” kinety, and the apical oral region (for Kentrophoridae, there are the degenerated anteriormost dikinetids) (Lynn 2008; Xu et al 2011a). According to Lynn 2008, Cryptopharyngidae and Loxodidae belong to the order Loxodida because of their laterally flattened, non-contractile cell, subapical oral region on the narrow ventral surface, and dikinetids surrounding the oral area as two perioral kineties and one intrabuccal kinety. However, according to the first molecular phylogenetic analysis on Cryptopharyngidae reported here, Cryptopharyngidae clusters with Kentrophoridae. Morphological support for this relationship includes the possession a layer of mucous or symbiotic bacteria on the left side and the laterally flattened body.

*Parakentrophoros canalis* grouped with *Kentrophoros* sp. (LT621835). Due to the lack of an accurate morphological identification of *Kentrophoro*s sp. (LT621835), this sequence may also belong to the new genus. Considering the two sequences are sister to *Cryptopharynx qingdaoensis* n. sp*.*, and *P. canalis* also possesses a degenerated subapical oral apparatus, *Parakentrophoros* is assigned to Cryptopharyngidae. The present study reports for the first time that the subapical buccal-like structure of Kentrophoridae (represented by *P. canalis*) possesses short kineties (perhaps vestiges of oral structures?). The subapical oral apparatus of Cryptopharyngidae is significantly more complex than the short kineties of *P. canalis.* Therefore, we posit that from Cryptopharyngidae to Kentrophoridae, the subapical oral apparatus has become progressively more degenerated (Fig. 2B).

### *Cryptopharynx qingdaoensis* n. sp.

#### Comparison with congeners (Fig. 13; Table 2)

**Table 2:** Comparison of *Cryptopharynx qingdaoensis* n. sp. with other species of *Cryptopharynx*

species	Length in vivo	RC number	IK number	Ma number	Data source
*C. qingdaoensis* n. sp.	75–110	15–22	7–12	1–2	This study
*C. minutus*	30–50	10–14	7–15	2–5	Xu et al. (2017)
*C. setigerus*	25–47^a^	6–11	3–4	2–3	Foissner (1995)
*C. mauritanicus*	110	> 40	–	50–70	Dragesco (1965)
*C. multinucleatum*	90	–	–	10–14	Dragesco (1954a, b, 1960)
*C. kahli*	135	–	–	–	Dragesco (1954a, b)
*C. enigmaticus*	70–100	17	–	1–5	Dragesco (1954a, b, 1960)
*Cryptopharynx* sp. 1	Ca. 50	15	Ca. 8	2	Foissner (1995)
*Cryptopharynx* sp. 2	130	22	Ca. 9	6	Foissner (1995)

^a^Data based on protargol-stained specimens. Measurements in μm; *IK* intrabuccal kinety, *Ma* macronucleus, *RC* right ciliary rows; – no data

Since the genus *Cryptopharynx* was established by Kahl (1928), nine species have been reported. They are easily distinguished from *C. qingdaoensis* n. sp. as follows.

*Cryptopharynx minutus* and *C. setigerus* differ from *C. qingdaoensis* n. sp. in having a smaller cell size (30–50 µm, 25–47 µm vs. 75–110 µm), fewer somatic kineties (10–14, 6–11 vs. 15–22) and more macronuclei (2–5, 2–3 vs. 1–2). Furthermore, there are fewer dikinetids in the intrabuccal kinety of *C. setigerus* than in *C. qingdaoensis* n. sp. (3–4 vs. 7–12) (Foissner 1995; Kahl 1928; Xu et al. 2017).

*Cryptopharynx mauritanicus* possesses more somatic kineties (> 40 vs. 15–22) and macronuclei (50–70 vs. 1–2) than *C. qingdaoensis* n. sp. (Dragesco 1965).

*Cryptopharynx multinucleatum* has far more macronuclei than *C. qingdaoensis* n. sp. (10–14 vs. 1–2 in the latter) (Dragesco 1954a, b, 1960).

*Cryptopharynx* sp. 2, which was reported by Foissner (1996), and *C. kahli* are both larger than *C. qingdaoensis* n. sp. (135 µm, 130 µm vs. 75–110 µm). Furthermore, *Cryptopharynx* sp. 2 has more macronuclei than *C. qingdaoensis* n. sp. (6 vs. 1–2) (Foissner 1996).

*Cryptopharynx enigmaticus* possesses button-shaped scales whereas *C. qingdaoensis* n. sp. possesses a mucous layer covered with irregular particles (Dragesco 1954a, b, 1960).

*Cryptopharynx* sp. 1, which was isolated by Foissner (1996), is significantly smaller than *C. qingdaoensis* n. sp. (50 µm vs. 80–120 µm).

### *Parakentrophoros* n. gen.

The new genus possesses a degenerated subapical oral apparatus which is similar to *Cryptopharynx*. Based on this character, *Parakentrophoros* n. gen. is tentatively assigned to the family Cryptopharyngidae. It is noteworthy, however, that *Parakentrophoros* n. gen. has a layer of symbiotic bacteria, a feature that has never been reported for Cryptopharyngidae. Kentrophoridae was established based on the presence of surface symbionts. Therefore, *Parakentrophoros* n. gen. may be an intermediate genus between the families Cryptopharyngidae and Kentrophoridae and may possibly represent a new family.

### *Parakentrophoros canalis* (Wright, 1982) n. gen. n. comb. (Fig. 14; Table 3, 4)

**Fig. 14:**
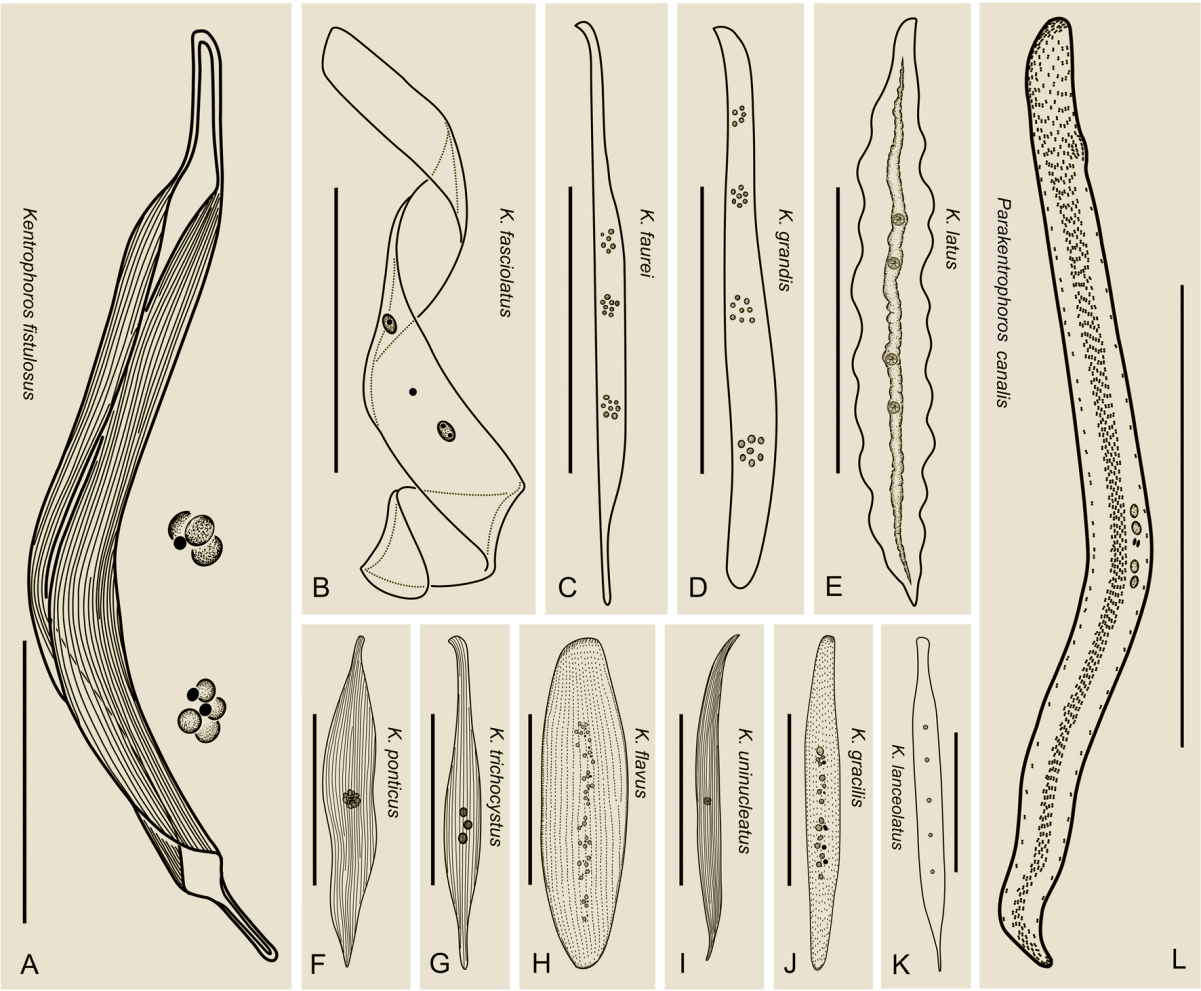
Illustrations of *Kentrophoros* and *Parakentrophoros* species. **A** *Kentrophoros fistulosus*, redrawn from Foissner (1995). **B** *Kentrophoros fasciolatus*, redrawn from Fauré-Fremiet (1950). **C** *Kentrophoros faurei*, redrawn from Dragesco (1954a, b). **D** *Kentrophoros grandis*, redrawn from Dragesco (1954a, b). **E** *Kentrophoros latus*, redrawn from Raikov (1962). **F** *Kentrophoros ponticus*, redrawn from Kovaleva (1966). **G** *Kentrophoros trichocystus*, redrawn from Dragesco (1954a, b). **H** *Kentrophoros flavus*, redrawn from Xu et al. (2011a). **I** *Kentrophoros uninucleatus*, redrawn from Raikov (1962). **J** *Kentrophoros gracilis.* **K** *Kentrophoros lanceolatus*, redrawn from Fauré-Fremiet (1951). **L** *Parakentrophoros canalis.* Scale bars: 300 μm (**C**, **D**, **E**, **H**, **J**, **L**); 200 μm (**A**, **B**, **K**, **F**, **I**); 100 μm (**G**)

**Table 3:** Comparison of species of *Kentrophoros* and *Parakentrophoros*

Species	Length in vivo	RC number	NG number	Ma per NG, number	Mi per NG, number	Length of bacteria	Data source
*P. canalis*	300–850	9–12	N	2–6	2	2.5	Wright (1982); this study
*K. fasciolatus*	100–1000	6–7	1	2	1	3	Fauré-Fremiet (1950); Sauerbrey (1928); Kahl (1933, 1935); Noland (1937)
*K. faurei*	750–1000	–	3	20–25	6	–	Carey (1992); Dragesco (1954a, b)
*K. fistulosus*	400–2800	23–43	2–34	2–9	1–4	5–15	Fauré-Fremiet (1950); Foissner (1995); This study
*K. flavus*	250–600	12–19	N	9–49	3–17	4	Raikov and Kovaleva (1968); Xu et al. (2011a)
*K. gracilis*	150–600	8–13	N	7–25	4–21	4–5	Raikov (1963); Xu et al. (2011a); This study
*K. grandis*	1000	–	4–6	27–30	10	–	Carey (1992); Dragesco (1954a, b)
*K. lanceolatus*	460–520	–	N	5–6	–	10	Fauré-Fremiet (1951)
*K. latus*	600–1200	–	1–4	4	2	5–6	Carey (1992); Raikov (1962)
*K. minutus*	170	Ca. 10	–	–	–	–	Dragesco (1960)
*K. ponticus*	300–400	17–20	1	6–12	6	–	Kovaleva (1966)
*K. trichocystus*	250	–	–	3	–	–	Dragesco (1954a, b)
*K. uninucleatus*	500–650	16–17	1	–	2	–	Raikov (1962)

Measurements in μm; *Ma* macronucleus, *Mi* micronucleus, *NG* nuclear group, *RC* right ciliary rows; N without this structure; – no data

**Table 4:** Comparison of morphometry of different *Parakentrophoros. canalis*, *Kentrophoros. fistulosus* and *K. gracilis* populations

species	Length in vivo	RC number	NG number	Ma per NG, number	Mi per NG, number	Length of bacteria	Data source
P. *canalis*	300–600	N	–	4–5	2	2.5	Wright (1982)
P. *canalis*	300–600	N	–	4–5	2	N	Carey (1992)
P. *canalis*	300–850	9–12	–	2–6	2	3	This study
*K. fistulosus*	700–2800	6–7*	17–34^a^	5–8	N	5^a^	Fauré-Fremiet (1950)
*K. fistulosus*	1000	N	N	N	N	N	Dragesco (1960)
*K. fistulosus*	~ 1000^a^	N	3–45	4–6	2–3	N	Kovaleva (1966)
*K. fistulosus*	N	N	N	N	N	N	Kovaleva and Golemansky (1979)
*K. fistulosus*	700–2800	N	~ 40	N	N	N	Carey (1992)
*K. fistulosus*	500–2000	30–43	10–30	3–9	1–4	5–15	Foissner (1995)
*K. fistulosus*	N	N	N	N	N	N	Azovsky and Mazei (2003a)
*K. fistulosus*	400–1500	23–40	2–20	2–4	1–2	5	This study
*K. gracilis*	300–350	12	–	7–10	4–6	6	Raikov (1963)
*K. gracilis*	350	N	–	N	N	N	Dragesco (1965)
*K. gracilis*	N	N	–	N	N	N	Kovaleva and Golemansky (1979)
*K. gracilis*	300–350	N	–	7–10	N	N	Carey (1992)
*K. gracilis*	N	N	–	N	N	N	Azovsky and Mazei (2003a)
*K. gracilis*	150–600	10–13	–	10–25	9–21	4–5	Xu et al. (2011a)
*K. gracilis*	200–400	8–11	–	7–19	4–13	6	This study

*Ma* macronucleus, *Mi*, micronucleus, *NG* nucler group, *RC* right ciliary rows; ^a^Data from illustration; N No data available; – Absent this structure; ^*^Doubtful

**Remarks and comparison:** *Parakentrophoros canalis* was first described by Wright (1982) under the name *Kentrophorose canalis*. In this study, the ciliature of this species was revealed for the first time. Based on its degenerated subapical oral apparatus, this species is transferred from *Kentrophoros* into *Parakentrophoros* n. gen. as *Parakentrophoros canalis* (Wright, 1982) nov. comb. (basionym *Kentrophoros canalis*). The original description was based solely on observations of specimens in vivo, therefore although the cell size and the number of macronuclei were recorded, details of the infraciliature were lacking (Wright 1982). Wright (1982) also reported that the apparent lightness of the central region of the cell is an effect produced by folding of the longitudinal cell margin extending towards, but not reaching, the central meridian of the body. However, based on careful observation of specimens both in vivo and following protargol staining, it can be seen that the transparent areas of the cell are caused by the absence of symbiotic bacteria. The Qingdao population closely resembles the original population in terms of these characters therefore its identity is not in doubt.

### ***Kentrophoros fistulosus ***(Fauré-Fremiet, 1950) Foissner, 1995 (Fig. 14; Table 3, 4)

According to Xu et al. (2011a), there are 15 species of *Kentrophoros.* However, *K. longissimus* (Dragesco, 1954) Foissner, 1995 and *K. tubiformis* Raikov & Kovaleva, 1966 are synonymized with *K. fistulosus* according to the similar cell size and number of nuclear groups (Foissner 1996). Furthermore, *K. canalis* is here transferred to *Parakentrophoros*. Therefore, *Kentrophoros* now contains only 12 species.

**Remarks and comparison:** This study is the first record of *K. fistulosus* in China. Before Foissner (1995) redescribed *K. fistulosus,* there were three species of *Kentrophoros* with more than 10 nuclear groups, namely *K. fistulosus*, *K. longissimus* and *K. tubiformis*. On first inspection, the main difference between *K. longissimus* and *K. fistulosus* is the cell shape (flattened ribbon-like vs. tubular). However, at low magnification (× 50), *K. fistulosus* also appears ribbon-like. Therefore, body shape alone is not sufficient to distinguish between these two species. An additional difference was thought to be the presence in *K. fistulosus* (vs. absence in *K. tubiformis)* of an envelope surrounding the individual nuclear groups (Dragesco 1954a, b; Raikov and Kovaleva 1966). However, ultrastructural evidence showed that *K. fistulosus* also lacks a nuclear envelope (Raikov 1972). Hence, *K. tubiformis* and *K. fistulosus* are synonyms. *Kentrophoros fistulosus* was first reported from Pouldohan, France, by Fauré-Fremiet (1950). The recorded length was 700–2800 µm and width 45 µm. The Chinese population resembles the original population except the number of right somatic kineties (23–40 vs. 6–7). Considering the tube-shape cell, this apparent difference may be due to the fact that only a small proportion of the total number of kineties was counted in the original description. Foissner (1995) redescribed this species based on a population from Roscoff, France. The size of this population is similar to the original population. Furthermore, the Roscoff population possesses 20–43 right somatic kineties, which is significantly more than that reported by Fauré-Fremiet (1950) for the original population, but similar to the Chinese population.

### ***Kentrophoros gracilis ***(Raikov, 1963) Foissner, 1995 (Fig. 14; Table 3, 4)

**Remarks and comparison:** *Kentrophoros gracilis* was first reported by Raikov (1963) with a detailed description of its morphometrics. The cell is 300–350 µm long in vivo and is ribbon-shaped. It possesses 12 right somatic kineties, 7–10 macronuclei and 4–6 micronuclei. The epibiontic bacteria cell length is 6 µm. Xu et al. (2011a) reported the first Chinese population, which resembles the original population in cell size and shape, and numbers of right somatic kineties, macronuclei, and micronuclei. The only nominal difference between them is the size of the epibiontic bacteria (4–5 µm vs. 6 µm). The present population is similar to the original population in cell size, numbers of macronuclei and micronuclei, and size of epibiontic bacteria, however it differs in the number of right somatic kineties (8–11 vs. 12). The number of right somatic kineties varies between and within populations, and those of the original and Qingdao populations both fall within this range of variability. Therefore, the Qingdao population is considered to be conspecific with *K. gracilis*.

## Conclusion

The present study of marine interstitial ciliates from a sandy beach at Qingdao, China, revealed one new species, *Cryptopharynx qingdaoensis* n. sp., one new genus, *Parakentrophoros* n. gen., a new combination, *Parakentrophoros canalis* (Wright, 1982) n. gen. n. comb., and a new record for Asia of *Kentrophoros fistulosus.* A redescription of *Kentrophoros gracilis,* based on the Qingdao population, is also provided. *Cryptopharynx qingdaoensis*, *Parakentrophoros canalis* and *Kentrophoros fistulosus* were sequenced for the first time. Cryptopharyngidae grouped within the Kentrophoridae assemblage. According to Lynn (2008), Cryptopharyngidae and Loxodidae are assigned to Loxodida. The taxonomic affiliation of Cryptopharyngidae therefore needs to be further investigated. With respect to the geographical distribution of Cryptopharyngidae, future sampling from global sandy marine environments will likely reveal expanded biogeographical ranges of known species and the discovery of new species. Furthermore, there remains a large number of known ciliate species adapted to marine interstitial environments that have yet to be sequenced. Expanded sampling and sequencing of such species will facilitate more robust phylogenetic analyses of the karyorelicteans.

## Data Availability

Four newly obtained SSU rDNA sequences were deposited in the GenBank database with accession numbers PQ113837–PQ113840. ZooBank registration number of the new genus: urn:lsid: zoobank.org:act:CABD9803-0E1F-4574-AFF2-A0547229A87C. ZooBank registration number of the new species: urn:lsid: zoobank.org:act:4D45D67B-7821-43C0-826C-A33A9B0BBCA1.
